# Pioglitazone does not synergize with mirabegron to increase beige fat or further improve glucose metabolism

**DOI:** 10.1172/jci.insight.143650

**Published:** 2021-03-22

**Authors:** Brian S. Finlin, Hasiyet Memetimin, Beibei Zhu, Amy L. Confides, Hemendra J. Vekaria, Riham H. El Khouli, Zachary R. Johnson, Philip M. Westgate, Jianzhong Chen, Andrew J. Morris, Patrick G. Sullivan, Esther E. Dupont-Versteegden, Philip A. Kern

**Affiliations:** 1 Division of Endocrinology, Department of Internal Medicine, College of Medicine,; 2 Barnstable Brown Diabetes and Obesity Center,; 3 Department of Physical Therapy and Center for Muscle Biology, College of Health Sciences,; 4 Spinal Cord and Brain Injury Research Center, College of Medicine,; 5 Department of Radiology, College of Medicine,; 6 College of Public Health, and; 7 Division of Cardiovascular Medicine, Department of Internal Medicine, College of Medicine, University of Kentucky, Lexington, Kentucky, USA.; 8 Lexington Veterans Affairs Medical Center, Lexington, Kentucky, USA.

**Keywords:** Clinical Trials, Metabolism, Adipose tissue, Drug therapy, Glucose metabolism

## Abstract

**BACKGROUND:**

Beige and brown adipose tissue (BAT) are associated with improved metabolic homeostasis. We recently reported that the β_3_-adrenergic receptor agonist mirabegron induced beige adipose tissue in obese insulin-resistant subjects, and this was accompanied by improved glucose metabolism. Here we evaluated pioglitazone treatment with a combination pioglitazone and mirabegron treatment and compared these with previously published data evaluating mirabegron treatment alone. Both drugs were used at FDA-approved dosages.

**METHODS:**

We measured BAT by PET CT scans, measured beige adipose tissue by immunohistochemistry, and comprehensively characterized glucose and lipid homeostasis and insulin sensitivity by euglycemic clamp and oral glucose tolerance tests. Subcutaneous white adipose tissue, muscle fiber type composition and capillary density, lipotoxicity, and systemic inflammation were evaluated by immunohistochemistry, gene expression profiling, mass spectroscopy, and ELISAs.

**RESULTS:**

Treatment with pioglitazone or the combination of pioglitazone and mirabegron increased beige adipose tissue protein marker expression and improved insulin sensitivity and glucose homeostasis, but neither treatment induced BAT in these obese subjects. When the magnitude of the responses to the treatments was evaluated, mirabegron was found to be the most effective at inducing beige adipose tissue. Although monotherapy with either mirabegron or pioglitazone induced adipose beiging, combination treatment resulted in less beiging than either alone. The 3 treatments also had different effects on muscle fiber type switching and capillary density.

**CONCLUSION:**

The addition of pioglitazone to mirabegron treatment does not enhance beiging or increase BAT in obese insulin-resistant research participants.

**TRIAL REGISTRATION:**

ClinicalTrials.gov NCT02919176.

**FUNDING:**

NIH DK112282 and P20GM103527 and Clinical and Translational Science Awards grant UL1TR001998.

## Graphical abstract

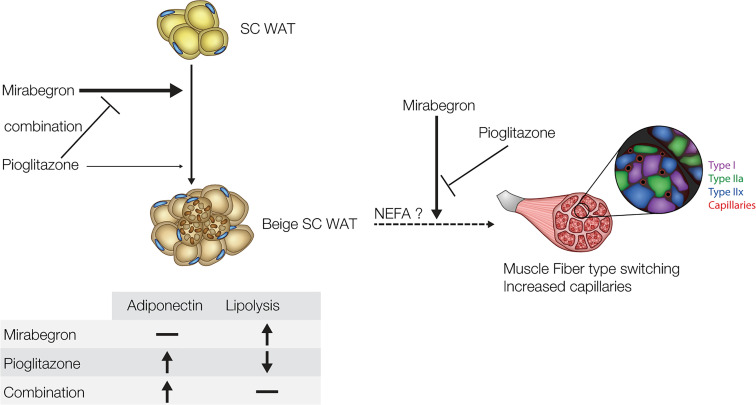

## Introduction

White adipose tissue (WAT) functions to store and release lipid and serves as an endocrine gland, secreting adipokines such as adiponectin and leptin to promote metabolic homeostasis (1). In obesity, white adipocytes become hypertrophied, followed by fibrosis, adipocyte necrosis, and immune cell infiltration, which leads to local and systemic inflammation, insulin resistance, and metabolic dysfunction (2–4). Furthermore, lipotoxicity occurs in tissues such as liver, skeletal muscle, and pancreatic β cells (5). In contrast to WAT, brown adipose tissue (BAT) consumes glucose and lipids to generate heat by uncoupled respiration mediated by uncoupling protein 1 (UCP1) (6), leading to improved glucose and lipid homeostasis. Although BAT is present in humans, it becomes much less prevalent with older age and obesity (recently reviewed in ref. 7). However, cold or β-adrenergic agonists cause the induction of UCP1-expressing adipocytes in WAT depots (reviewed in ref. 6) called beige adipocytes, which are unique since they have a different developmental origin than brown adipocytes (6, 8–10). Beige adipocytes are also associated with improved metabolic homeostasis in rodents (11–14), and the fact that they are highly inducible makes them, along with BAT, an attractive target for combating metabolic disease. Indeed, our recent studies in humans found that mirabegron increased beiging, and this was accompanied by improved glucose homeostasis (15, 16).

BAT and beige adipose tissue cause metabolic benefits, and research efforts have thus been aimed at identifying and understanding mechanisms that control browning and beiging. Cold is recognized as a potent stimulus for increasing human brown fat activity, and many studies have reported improvement in insulin sensitivity, and glucose and lipid homeostasis, after cold treatment (17–25). Some of those studies were performed on obese research participants and found that cold induced BAT and improved metabolism (20, 22, 24, 26). Cold also increased beiging of human subcutaneous WAT in lean and obese subjects (16), and a long list of other factors, identified mostly from studies in rodents, induce or inhibit adipose beiging (27), including control by the immune system (28, 29) as well as conditions that result in high catecholamine levels such as pheochromocytoma, cancers, and burns (30–32). Whether results from those studies can be translated into therapeutics remains to be determined.

Mirabegron and pioglitazone are 2 drugs that could be repurposed to induce beiging of WAT or increase BAT activity. After many unsuccessful past attempts to develop β_3_-adrenergic receptor (β_3_AR) agonists (33), mirabegron, a relatively specific β_3_ agonist, was approved for the treatment of overactive bladder and has been demonstrated to stimulate BAT and to induce beiging in subcutaneous (SC) WAT (15, 16, 34–36). Importantly, 2 studies showed that mirabegron treatment improved glucose homeostasis and increased both insulin sensitivity and β cell function (15, 34). These findings, along with the observation that mirabegron treatment stimulated lipolysis and caused SC WAT remodeling, suggest that mirabegron treatment caused changes in SC WAT that play a central role in the improvement in glucose homeostasis (15, 34, 37).

Thiazolidinediones (TZDs) are PPARγ agonists that are potent insulin sensitizers and have been shown to increase BAT (38, 39) and beige adipocytes (40, 41) in rodents and in vitro (42–44). The TZD rosiglitazone was shown to increase UCP1 in WAT in rodents (45), and pioglitazone treatment was subsequently shown to increase *UCP1* mRNA in SC WAT in humans (46, 47). Furthermore, some studies demonstrated synergism between TZDs and β_3_AR agonists in vivo in rodents (48, 49) or in vitro (43). Since both TZDs and β_3_AR agonists increase beiging or browning through different mechanisms, there is a strong rationale for assessing the ability of the combination of a TZD and β_3_AR agonist to induce beige adipose tissue and BAT in humans.

This study was designed to determine whether pioglitazone, a PPARγ activator, would work in an additive or synergistic manner with mirabegron, a β_3_AR agonist, to stimulate BAT or beige fat and therefore further improve glucose and lipid metabolism. Secondary objectives were to evaluate the underlying mechanisms for potential changes in these processes. We treated obese subjects with pioglitazone (30 mg/d) or a combination of pioglitazone (30 mg/d) and mirabegron (50 mg/d) and compared the results of these treatments with our recently published results of treatment with mirabegron alone (15).

## Results

### Study design, baseline characteristics of the research participants, and treatment responses.

**Figure 1:**
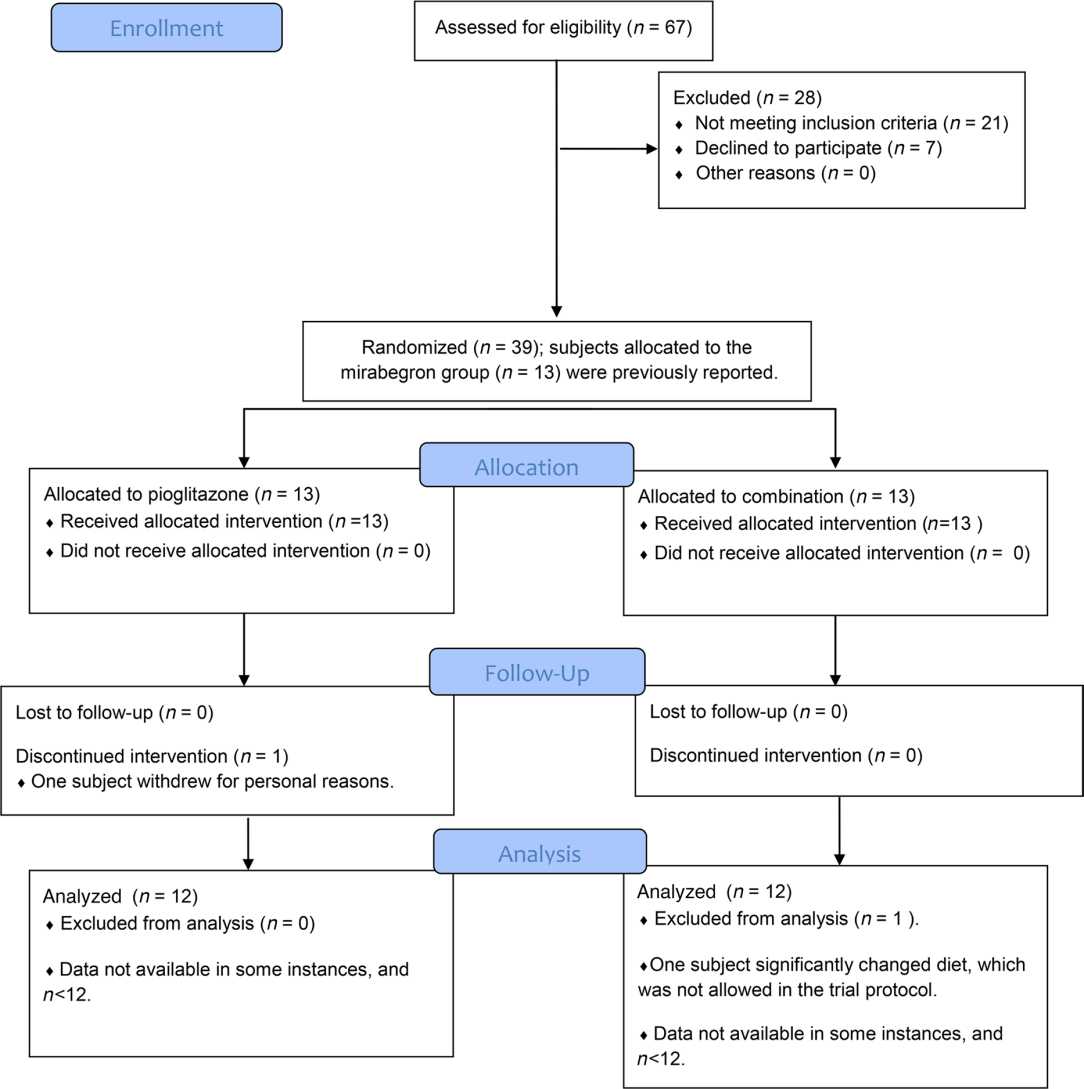
Flow chart of the study. A total of 67 subjects were assessed for eligibility to participate in the study, and 39 were allocated into 3 treatment groups (*n* = 13). Data from subjects in the mirabegron treatment group have been previously reported (15, 16). One subject withdrew from the study in the pioglitazone treatment group, and 1 subject was excluded from analysis in the combination treatment group.

This study was designed to evaluate whether combination therapy with mirabegron and pioglitazone, at doses that are FDA approved for their respective indications, increases beige adipose tissue or BAT more than either drug alone. The results of combination treatment and monotherapy with pioglitazone are presented and analyzed with the results of monotherapy with mirabegron, which has been recently published (15, 16). Secondary outcomes were glucose and lipid homeostasis, insulin sensitivity, β cell function, changes in muscle fiber type composition, and characterization of SC WAT gene expression. A flow diagram showing the overall study design is shown in Figure 1. Some of the results from the mirabegron-only treatment group have already been published (15, 16). The baseline characteristics and treatment responses of the subjects in the pioglitazone (30 mg/d) and combination (mirabegron and pioglitazone) treatment groups are shown in Table 1, and the data from the mirabegron treatment groups, which were previously published (15), are shown in Supplemental Table 1; supplemental material available online with this article; https://doi.org/10.1172/jci.insight.143650DS1 The groups were generally well matched, although weight (but not BMI) was slightly higher in the combination treatment group than in the mirabegron treatment group (*P* = 0.04). There was no significant change in body weight after treatment. Lean and fat mass were evaluated by dual-energy x-ray absorptiometry (DEXA), and pioglitazone caused an increase in fat mass, as expected (50) (Supplemental Table 2). Neither treatment affected energy expenditure, which was evaluated by indirect calorimetry (Supplemental Table 3). Pioglitazone treatment caused a small, but significant, increase in heart rate, and combination therapy caused a small, but significant, increase in diastolic blood pressure (Table 1). Pioglitazone treatment significantly decreased cholesterol, and there was a trend for decreased HbA1c (*P* = 0.09) and TG (*P* = 0.08) (Table 1). Combination therapy significantly decreased 120-minute glucose and HbA1c and increased HDL-cholesterol (Table 1). We further determined whether changes caused by the treatments were significantly different from each other (changes in response to mirabegron treatment were calculated using previously published data; refs. 15, 16). This analysis revealed no significant differences in treatment responses (Supplemental Table 4), and it is notable that in response to mirabegron, pioglitazone, or the combination, similar treatment responses were observed in fasting glucose, HbA1c, or fasting lipids (Table 1, Supplemental Table 4, and ref. 15).

### Beige and brown adipose tissue.

**Figure 2:**
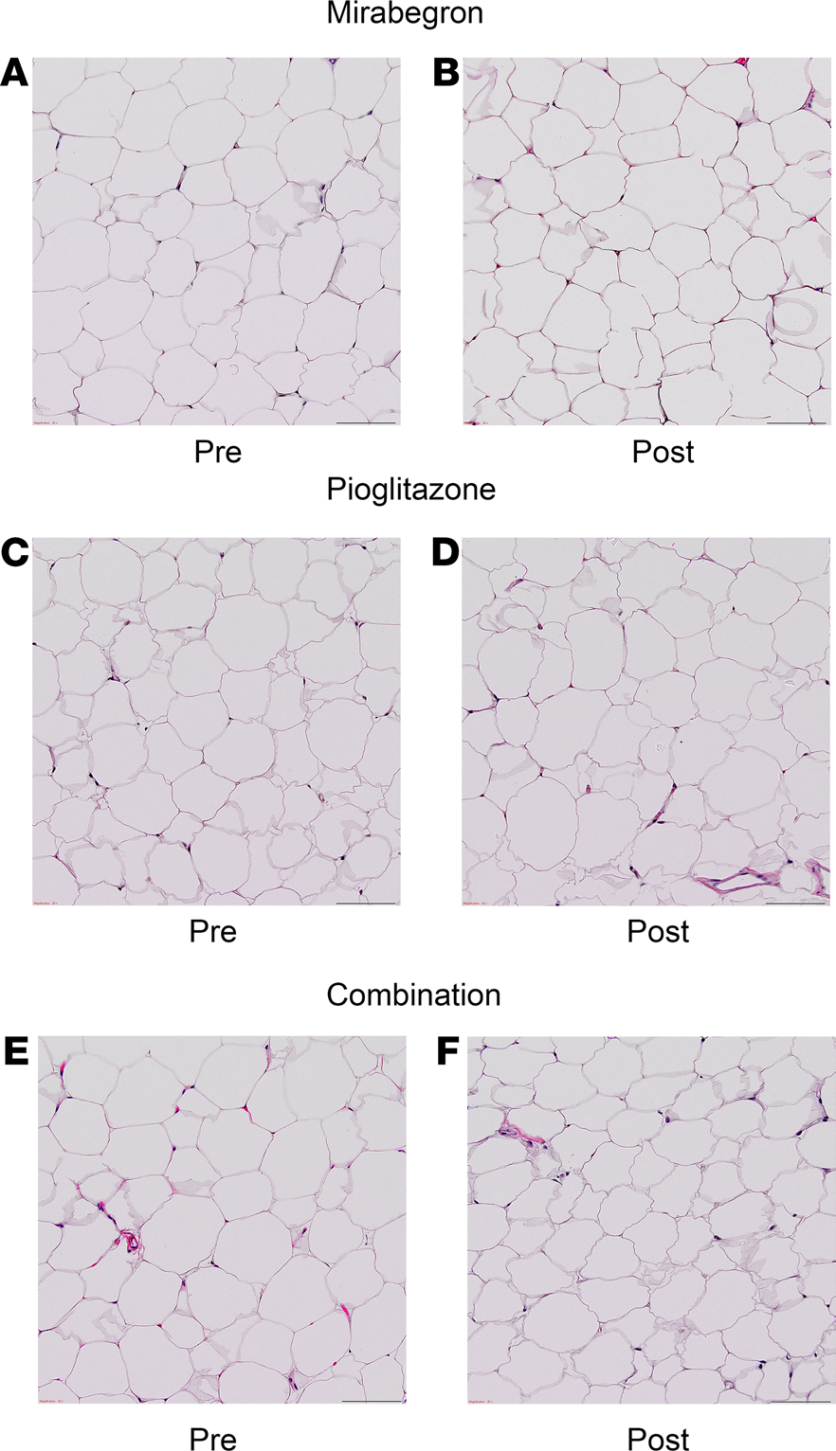
Hematoxylin and eosin staining of SC WAT. Representative images of SC WAT stained with hematoxylin and eosin are shown for (**A** and **B**) mirabegron, (**C** and **D**) pioglitazone, and (**E** and **F**) combination. Scale bars: 100 μm.

**Figure 3:**
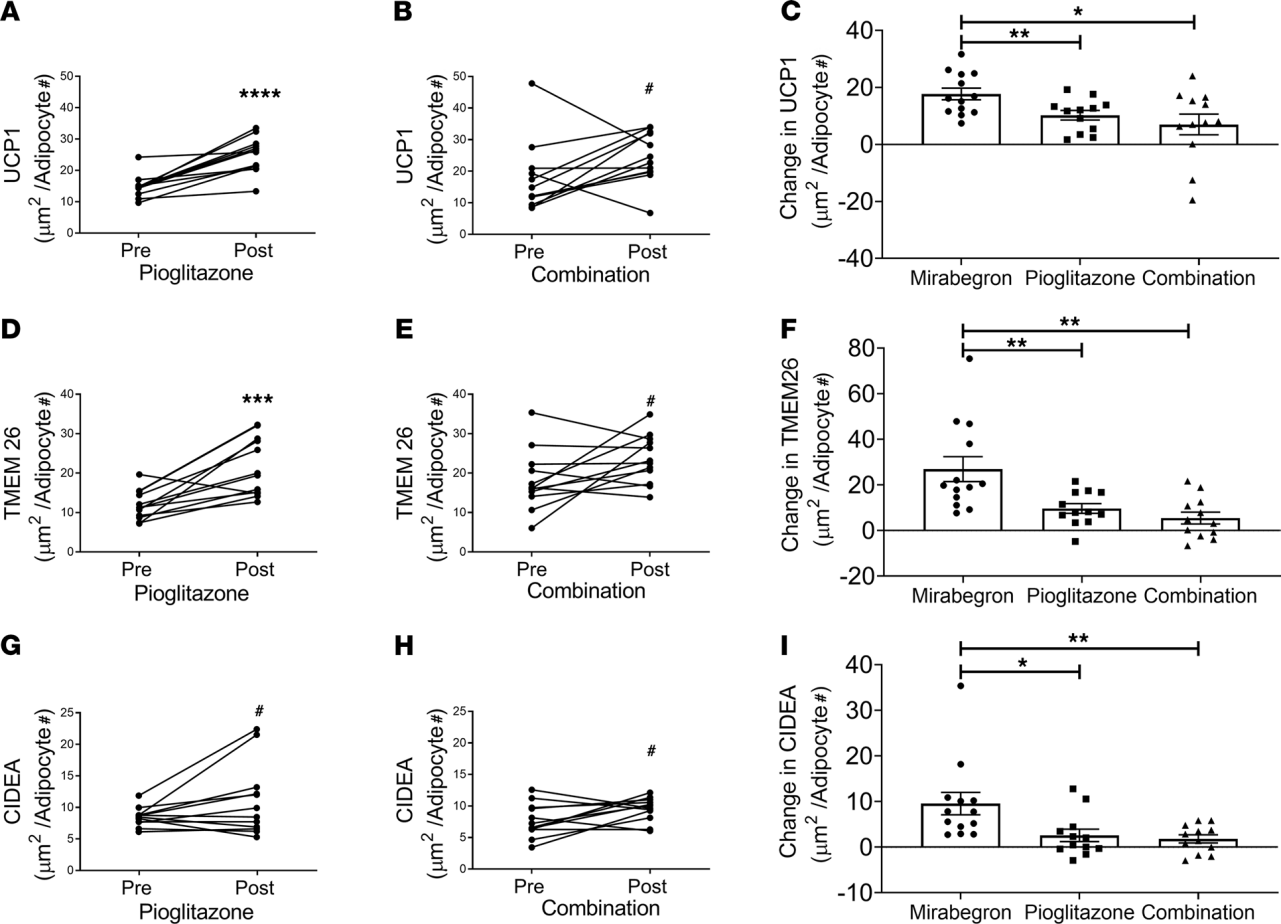
Combination treatment does not increase SC WAT beiging more than mirabegron treatment. The protein levels of 3 markers of WAT beiging were determined in SC WAT by immunohistochemistry before and after treatment as described in Methods. The change in protein caused by mirabegron treatment (*n* = 13) was calculated using previously published data (15, 16); see Supplemental Figure 4. (**A** and **B**) Analysis of UCP1 expression: data from the pioglitazone (*n* = 12) and combination treatment groups (*n* = 12) were analyzed by 1-way ANOVA with data from the mirabegron treatment group as described in Methods. (**C**) Analysis of the change in UCP1 caused by the 3 treatments (interaction *P* = 0.01); the change (post-pre treatment) in protein was calculated and analyzed by 1-way ANOVA as described in Methods (**P < 0.01; *P < 0.05). (**D**–**F**) Analysis of TMEM26 expression (**F**: interaction *P* = 0.01). (**G**–**I**) Analysis of CIDEA expression (**I**: interaction *P* = 0.02). The data in (**D**–**I**) were analyzed as described for **A**–**C**. Data in **C**, **F**, and **I** indicate the mean ± SEM; ****P < 0.0001; ***P < 0.001; **P < 0.01; *P < 0.05; ^#^*P* < 0.1.

**Figure 4:**
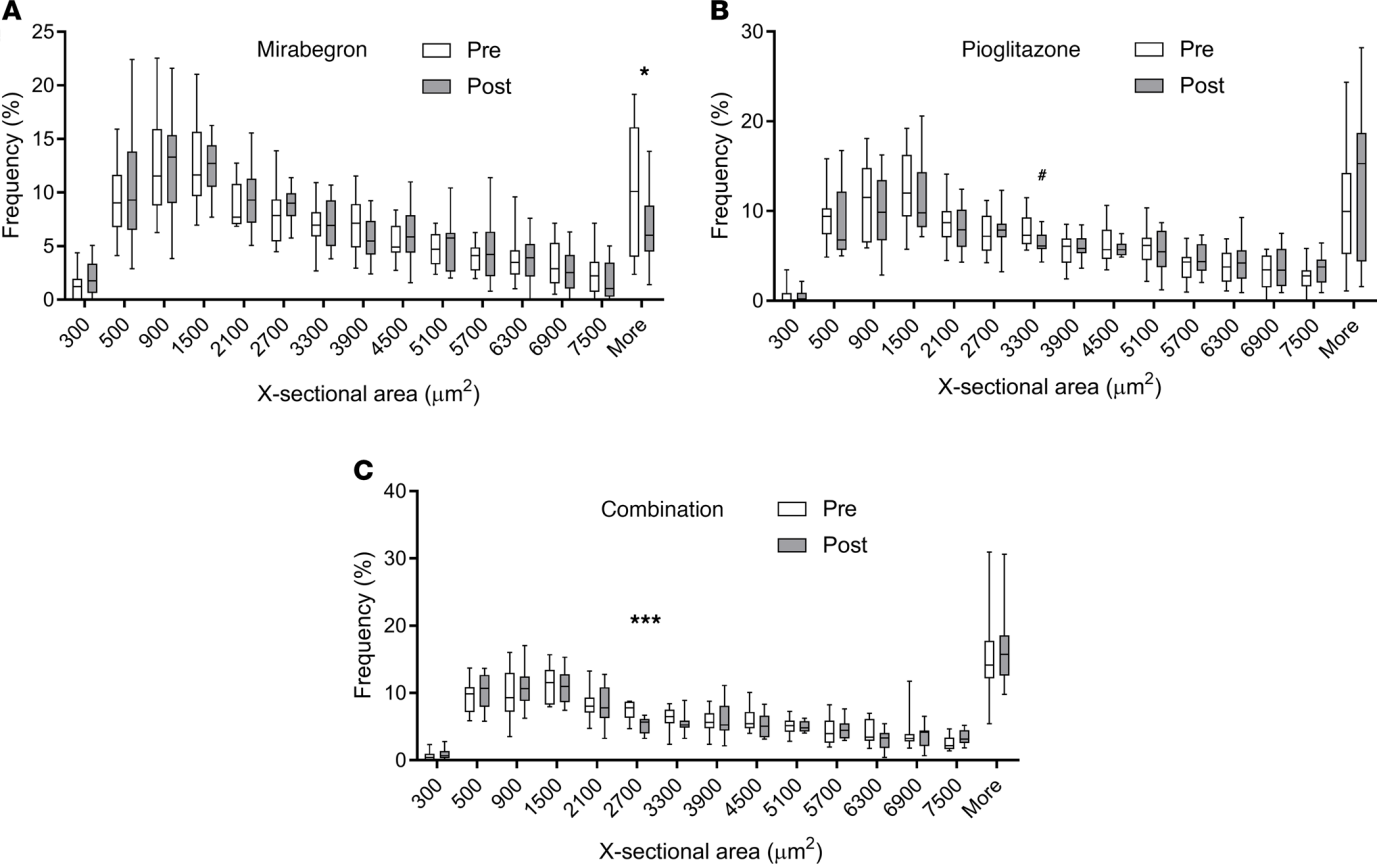
Quantification of adipocyte size. Adipocyte size was quantified in the mirabegron (**A**), pioglitazone (**B**), and combination (**C**) treatment groups as described in Methods. Histograms were generated using the percentage of adipocytes in the indicated bins, and the data indicate mean ± SEM for the number of adipocytes in each bin before and after mirabegron (*n* = 13), pioglitazone (*n* = 12), and combination treatment (*n* = 12) and were analyzed by a paired, 2-tailed Student’s *t* test; *P ≤ 0.05; ***P ≤ 0.1; ^#^*P* ≤ 0.10.

**Figure 5:**
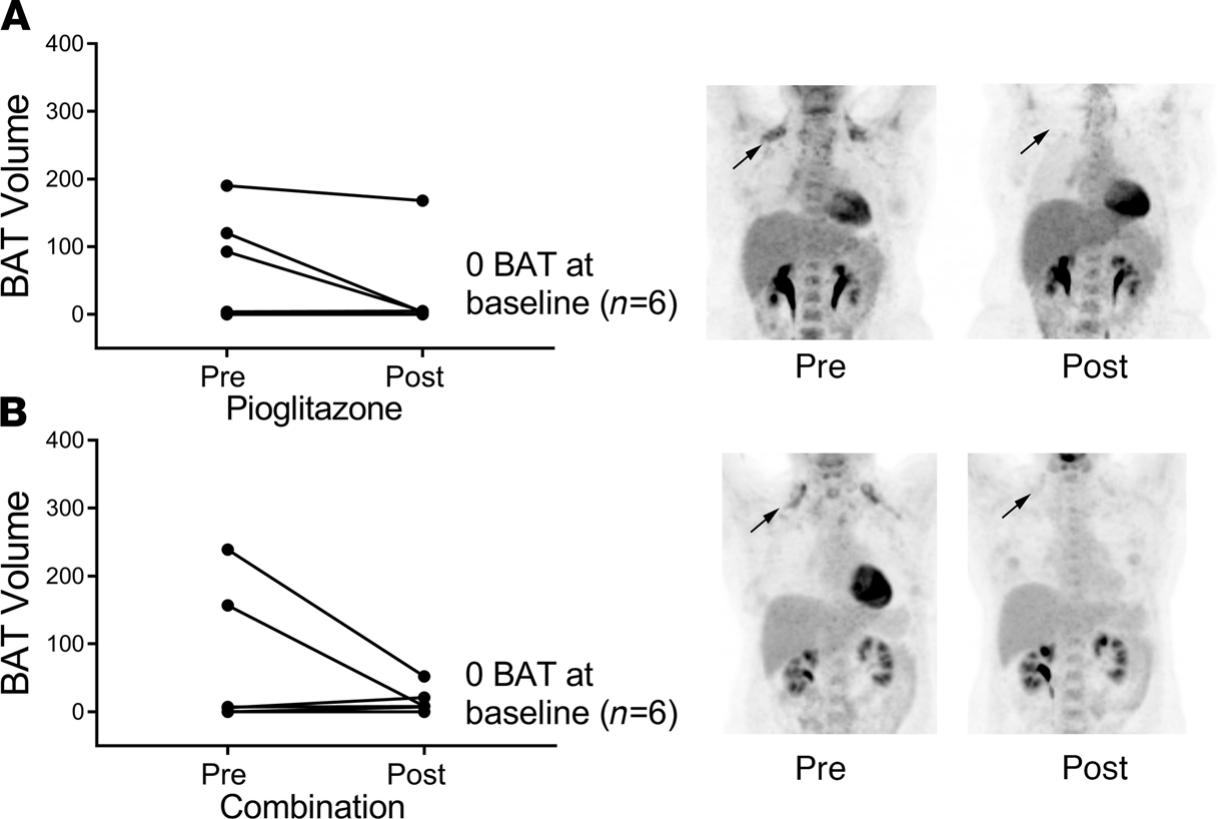
Neither pioglitazone nor combination treatments induce BAT in obese, insulin-resistant research participants. The volume of BAT was quantified by PET CT scans before and after treatment with (**A**) pioglitazone (*n* = 10) or (**B**) the combination of pioglitazone with mirabegron (*n* = 10). The number of subjects that had no detectable BAT at baseline is shown. Arrows indicate the supraclavicular region. Data were analyzed by a paired, 2-tailed Student’s *t* test.

Since PPARγ activators such as pioglitazone increase beige and brown fat (39–41, 46, 47, 51), our primary goal was to determine whether combination treatment increased beige or brown adipose tissue more than pioglitazone or mirabegron alone. Using immunohistochemistry, we characterized adipose beiging by determining the protein expression of UCP1, transmembrane protein 26 (TMEM26), and cell death inducing DFFA like effector a (CIDEA) in SC WAT before and after treatment. Hematoxylin and eosin staining of SC WAT to show the adipose tissue morphology is shown in Figure 2; we did not observe significant changes in the morphology or fields of multilocular adipocytes after any of the treatments. Representative images of immunohistochemical staining for UCP1, TMEM26, and CIDEA before and after each of the 3 drug treatments is shown in Supplemental Figures 1–3, respectively. The pattern of staining was complex. In addition to a crescent-shaped pattern around adipocytes, we detected staining of smaller cells between adipocytes, which could represent beige adipocytes. However, we have observed previously that some of the UCP1 staining colocalizes with CD163 macrophages (15). The data for mirabegron treatment group were previously published (15, 16), and the quantified data are available in Supplemental Figure 4. Pioglitazone significantly increased UCP1 expression (Figure 3A; *P* < 0.0001), and combination treatment caused a trend for an increase (Figure 3B; *P* < 0.1). To determine whether there were differences in the magnitude of response caused by mirabegron, pioglitazone, or combination treatment on UCP1 induction, we calculated the change in UCP1 (post-pre), using previously published data to calculate the change caused by mirabegron treatment (15, 16). This analysis revealed that there was a significant difference in the induction of UCP1 in response to the different treatments (interaction *P* = 0.01). Mirabegron treatment alone was more effective than pioglitazone at inducing UCP1 (Figure 3C; *P* < 0.01). Despite the fact that both mirabegron and pioglitazone significantly induced UCP1, the addition of pioglitazone to mirabegron treatment resulted in less UCP1 induction than mirabegron alone (mirabegron versus combination: *P* < 0.05). Similar results were obtained when we analyzed the expression of the beige adipose marker TMEM26; pioglitazone treatment significantly induced TMEM26 (*P* < 0.001), combination caused a trend for an increase (*P* < 0.1), and combination treatment resulted in less TMEM26 induction than mirabegron alone (Figure 3, D–F). Neither pioglitazone nor combination treatment significantly induced CIDEA expression, although there was a trend with both treatments (Figure 3, G and H). We previously observed that mirabegron treatment induced CIDEA (15), and this response caused by mirabegron treatment was significantly different than pioglitazone or combination therapy (Figure 3I; interaction *P* = 0.02), with mirabegron increasing CIDEA more than pioglitazone (*P* < 0.05) or combination treatments (*P* < 0.01). Together, these results indicate that mirabegron treatment induced SC WAT beiging more than pioglitazone or combination treatments and that the addition of pioglitazone to mirabegron treatment attenuated the beiging response of mirabegron treatment. We did not observe differences between pioglitazone and combination treatments in the induction of beige marker protein expression; a limitation of this study is that it did not have sufficient power to detect small changes given the significant variability in baseline expression levels of the marker proteins. As noted above, there were no changes in body weight with any of the treatments. None of the treatments caused substantial changes in adipocyte size (Figure 4), although mirabegron treatment reduced the size of very large adipocytes (*P* = 0.05), which could represent important adipose tissue remodeling. We previously reported that mirabegron at 50 mg/d failed to increase BAT volume in obese, insulin-resistant research participants (15). Notably, the majority of subjects in that study (8 of 13) had no detectable BAT at baseline, and mirabegron treatment did not result in the appearance of detectable in BAT in those 8 subjects (15). In subjects treated with pioglitazone, there was no significant increase in BAT volume or BAT activity (Figure 5A and Supplemental Table 5). Indeed, in 6 of 12 subjects who had no BAT at baseline, no increase in BAT was detected. Notably, BAT volume decreased in the 3 subjects with detectable BAT at baseline (Figure 5A), but it would require a larger study of subjects with preexisting BAT to determine definitively whether pioglitazone significantly reduces BAT volume. The combination of mirabegron and pioglitazone also did not induce BAT (Figure 5B). Similar to the cohort of pioglitazone-treated subjects, 6 of 12 subjects had no BAT at baseline and did not exhibit an increase.

### Glucose homeostasis.

**Figure 6:**
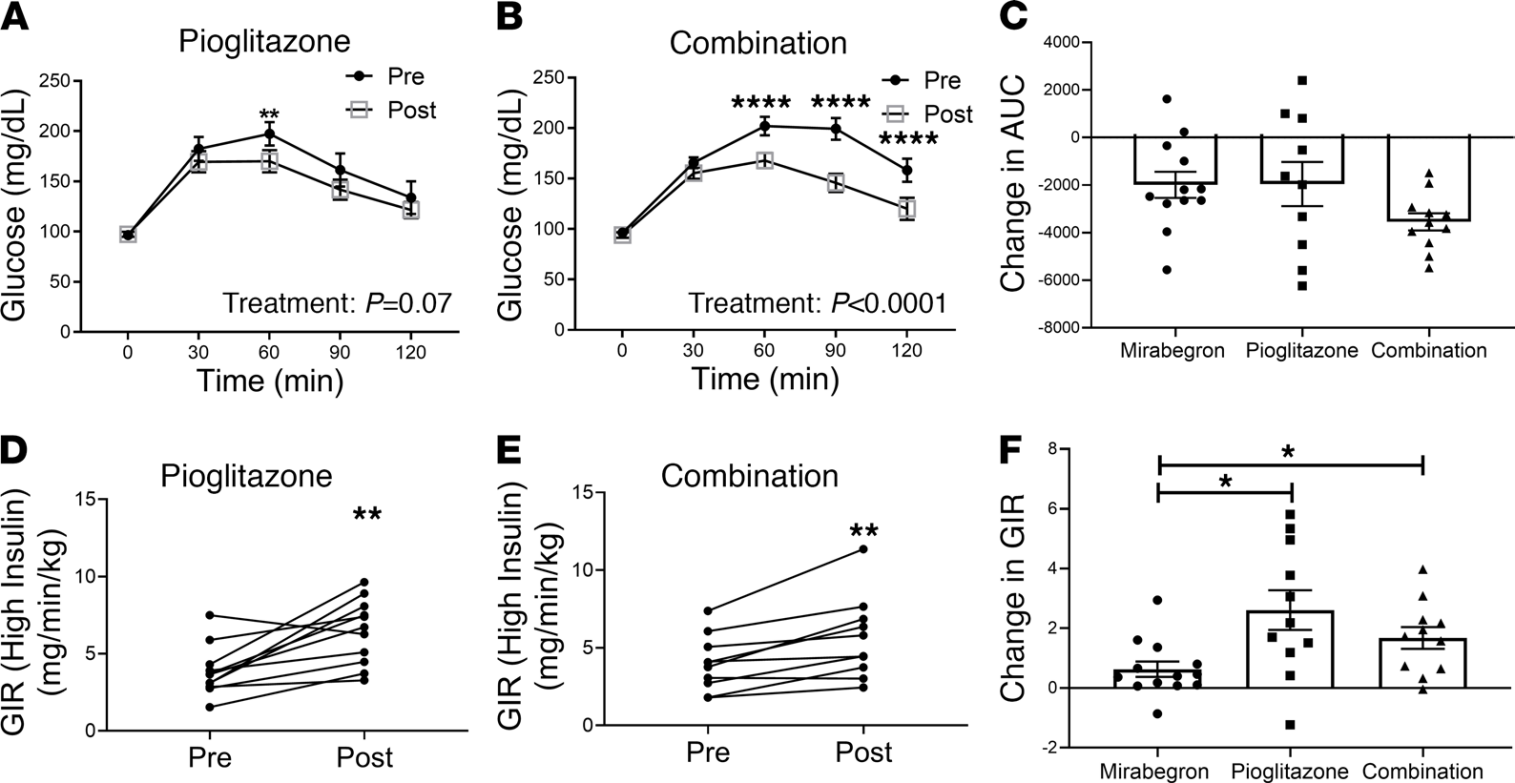
Evaluation of oral glucose tolerance and insulin sensitivity in response to pioglitazone and combination treatments. Subjects were treated with pioglitazone (30 mg/d) or a combination of pioglitazone (30 mg/d) and mirabegron (50 mg/d) for 12 weeks. Oral glucose tolerance was assessed at baseline and at the end of treatment in the (**A**) pioglitazone (*n* = 10) and (**B**) combination (*n* = 11) treatment groups. Data indicate mean ± SEM and were analyzed by repeated measures 2-way ANOVA. **P < 0.01; ****P < 0.0001. (**C**) The AUC was determined and then the change (post-pre) for the 3 different treatments was calculated. Data indicate the mean ± SEM and were analyzed by ANOVA with previously published data from the mirabegron treatment group (15) as described in Methods (interaction *P* = 0.06). (**D** and **E**) Insulin sensitivity was assessed by euglycemic clamping at an insulin infusion rate of 1 mU/kg/min and determining glucose infusion rate (GIR) before and after treatment. Data were analyzed by 1-way ANOVA with previously published data from the mirabegron treatment group (15) as described in Methods. Pioglitazone (*n* = 11); combination (*n* = 10); **P < 0.01. (**F**) The change in GIR (post-pre) caused by each treatment was calculated. Data indicate the mean ± SEM and were analyzed by 1-way ANOVA with previously published data from the mirabegron treatment group (15) as described in Methods (interaction *P* = 0.02); *P < 0.05.

We determined whether the combination of mirabegron and pioglitazone would differ from pioglitazone alone and compared each with previously published data on the effects of mirabegron (15). We performed a standard 75 g oral glucose tolerance test (OGTT); there was a trend for improved glucose tolerance after pioglitazone treatment (Figure 6A), and combination treatment significantly improved oral glucose tolerance (Figure 6B). To determine whether there were significant differences in the magnitude of the response caused by the 3 different treatments, we calculated the area under the curve (AUC) and analyzed the change in treatment responses (post-pre). There was a trend (interaction: *P* = 0.06) for a greater decrease in AUC caused by combination treatment than monotherapy with pioglitazone or mirabegron (Figure 6C). An additive effect of the 2 drugs on glucose tolerance is an interesting possibility that would need to be explored in a larger study. We performed euglycemic clamping to measure insulin sensitivity, and as expected, pioglitazone alone (Figure 6D) or in combination with mirabegron (Figure 6E) significantly improved insulin sensitivity. We compared the magnitude of change by the 3 drug treatments and found that both pioglitazone and combination treatments resulted in a greater increase in insulin sensitivity than mirabegron treatment (Figure 6F; interaction: *P* = 0.02; pioglitazone versus mirabegron: *P* = 0.02; combination versus mirabegron: *P* = 0.05). Finally, we calculated the disposition index, a measure of insulin secretion adjusted for insulin sensitivity (52), as the product of the insulinogenic index and the glucose infusion rate. Neither pioglitazone nor combination treatment caused a significant change in the disposition index calculated using either the glucose infusion rate or the Matsuda index (not shown) as measures of insulin sensitivity, and we did not detect a significant difference in treatment responses (Supplemental Table 4).

### Muscle fiber type switching and lipotoxicity.

**Figure 7:**
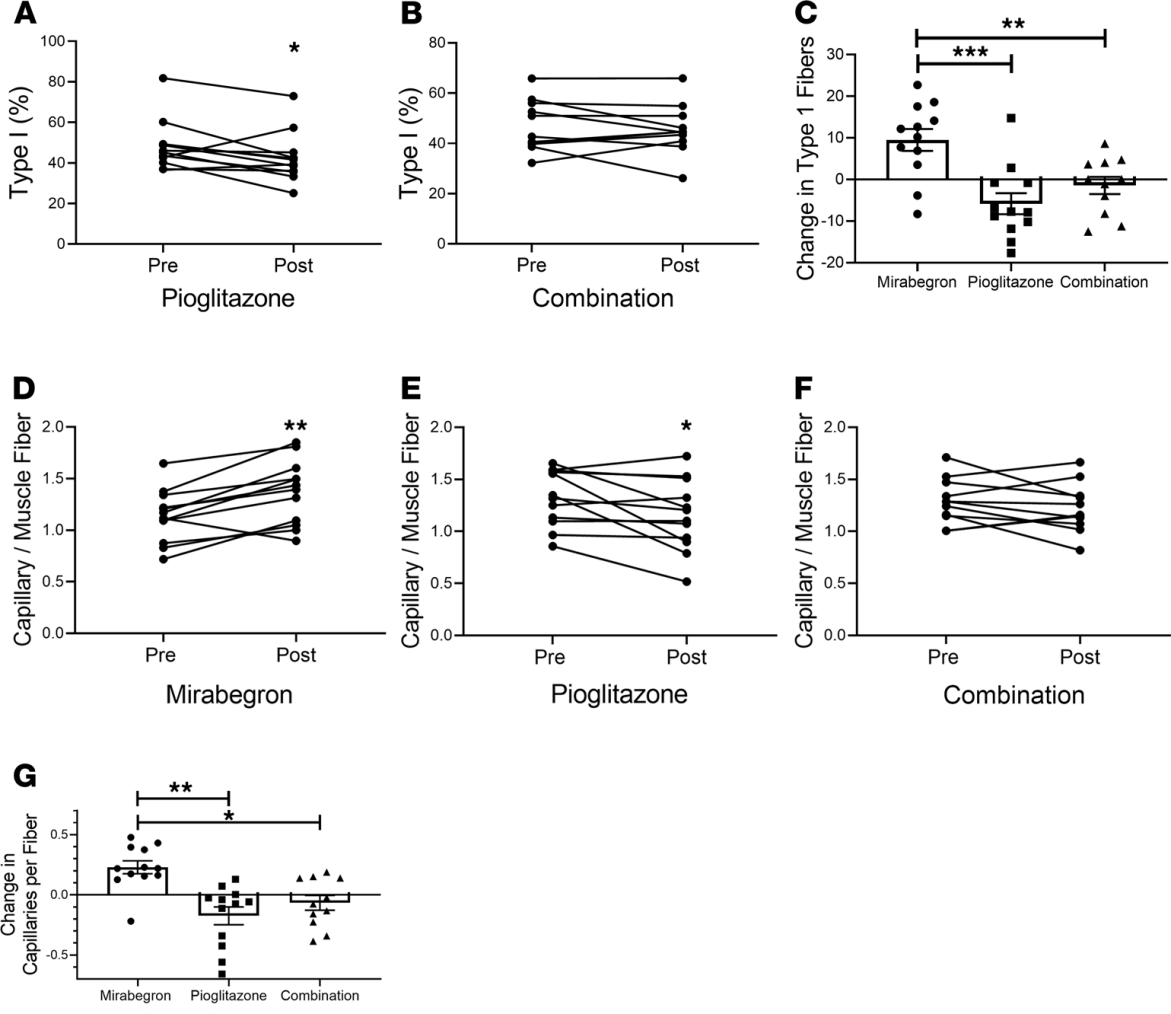
Evaluation of fiber type switching in vastus lateralis muscle. (**A**) The percentage of type I fibers before and after pioglitazone treatment (*n* = 12). (**B**) The percentage of type I fibers before and after combination treatment (*n* = 11). Data were analyzed by 1-way ANOVA with previously published data from the mirabegron treatment group (15) as described in Methods. (**C**) The change in type 1 fibers (post-pre) caused by each treatment was calculated and analyzed by 1-way ANOVA with previously published data from the mirabegron treatment group (15) as described in Methods (interaction *P* = 0.001); data indicate mean ± SEM; **P < 0.01; ***P < 0.001. (**D**–**F**) Capillaries per muscle fiber were quantified before and after treatment with mirabegron (*n* = 12), pioglitazone (*n* = 12), or mirabegron and pioglitazone (*n* = 11). (**G**) The change in capillaries per fiber was calculated; data indicate mean ± SEM and were analyzed by 1-way ANOVA as described in Methods (interaction *P* < 0.001); *P < 0.05; **P < 0.01.

We previously observed that mirabegron treatment increased type I fibers in vastus lateralis muscle and lowered muscle TG levels (15). Type I muscle fibers are more oxidative and are typically observed in subjects who are insulin sensitive (53, 54), suggesting that fiber type switching might be a mechanism for the increase in insulin sensitivity by mirabegron treatment as previously published (15). Herein we wanted to test whether the increase in insulin sensitivity with pioglitazone and the combination treatment were related to changes in muscle fiber type. Pioglitazone treatment decreased type I fibers (Figure 7A; *P* < 0.05), and combination treatment had no effect (Figure 7B). Thus, there was a significant difference in the change observed for type I fibers in response to the different treatments (Figure 7C), such that they were increased by mirabegron, decreased by pioglitazone, and unchanged by the combination of 2 drugs, essentially cancelling out the effect of the other. We also measured type IIa and type IIx fibers in vastus lateralis but did not find significant changes caused by either pioglitazone or combination treatments (Supplemental Figure 5).

Since increased blood flow to muscle could be part of the mechanism for increased insulin sensitivity caused by these different treatments (55, 56), we measured muscle capillary density. Mirabegron treatment significantly increased capillary density (Figure 7D), whereas capillary density decreased in response to pioglitazone (Figure 7E), and combination treatment resulted in no change (Figure 7F). Mirabegron and pioglitazone had significant opposite effects on muscle capillary density (Figure 7G). Thus, in terms of both fiber switching and changes in capillary density, pioglitazone and mirabegron treatment have different effects on muscle.

**Figure 8:**
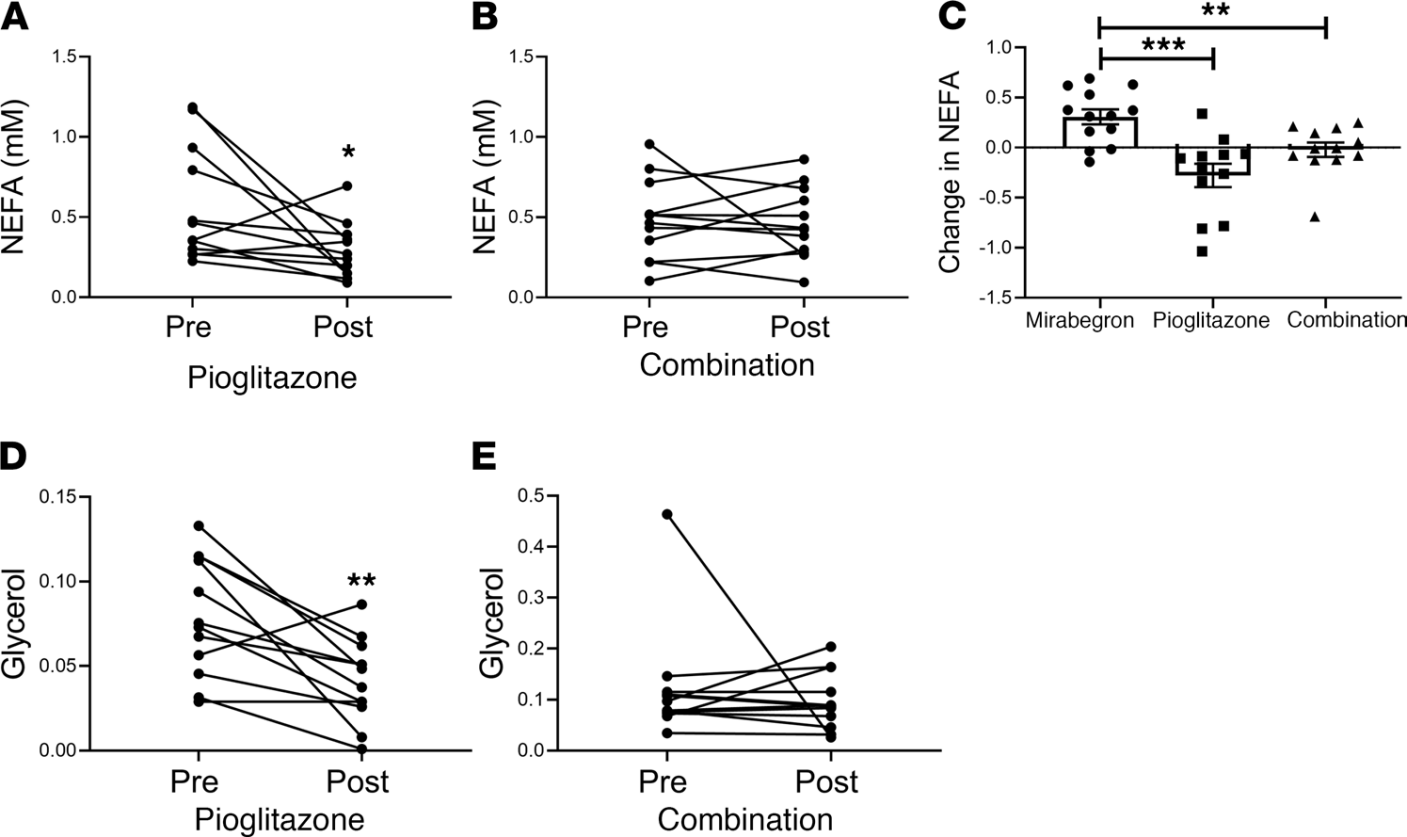
Evaluation of plasma NEFA and glycerol. Plasma NEFA levels before and after treatment with (**A**) pioglitazone (*n* = 12) or (**B**) combination (*n* = 12) are shown. Data were analyzed by 1-way ANOVA with previously published data from the mirabegron treatment group (15) as described in Methods; *P < 0.05. (**C**) The change in NEFA (post-pre) caused by each treatment was calculated and analyzed by 1-way ANOVA as described in Methods (interaction *P* = 0.001); data indicate mean ± SEM; **P < 0.01; ***P < 0.001. Plasma glycerol levels before and after treatment with (**D**) pioglitazone (*n* = 12) or (**E**) pioglitazone and mirabegron (*n* = 12) are shown. Data were analyzed by 1-way ANOVA with previously published data from the mirabegron treatment group (15) as described in Methods; **P < 0.01.

Mirabegron is a β_3_AR agonist and stimulates adipose lipolysis and plasma nonesterified fatty acid (NEFA) levels (16); however, this did not result in increased muscle ceramide or diacylglycerol levels (15). In subjects treated with pioglitazone, there was a reduction in plasma NEFA, but combination treatment with mirabegron and pioglitazone had no significant effect on NEFA (Figure 8, A and B). The change in plasma NEFA caused by mirabegron, pioglitazone, or combination treatments was significantly different (Figure 8C), demonstrating opposite effects of these drugs and the effective cancellation of the changes in NEFA in the combination group. Similarly, pioglitazone treatment reduced plasma glycerol, but combination treatment had no significant effect (Figure 8, D and E). However, when we analyzed whether the magnitude of the treatment responses were different, we did not detect a significant difference between the 3 treatment groups (interaction *P* = 0.89). To determine whether the reduction in NEFA by pioglitazone treatment reduced lipotoxicity, we measured TG, ceramide, and diglyceride levels in the vastus lateralis muscle biopsies but did not find significant differences caused by either pioglitazone or combination treatments (Supplemental Figure 6).

### Systemic inflammation.

**Figure 9:**
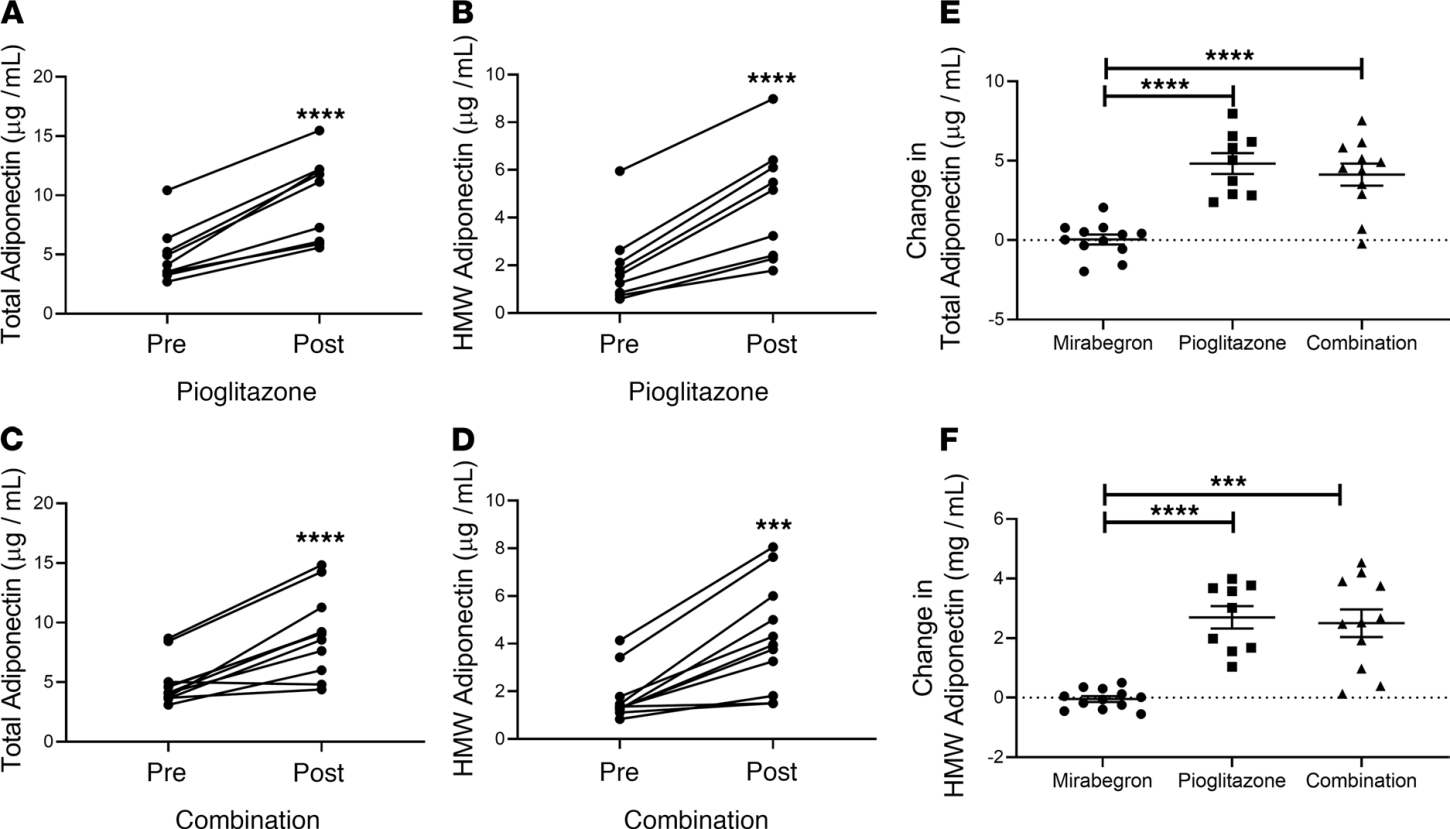
Evaluation of total and HMW adiponectin. (**A**–**D**) Plasma total and HMW adiponectin levels before and after treatment with pioglitazone (*n* = 9) or pioglitazone and mirabegron (*n* = 11) are shown. Data were analyzed by 1-way ANOVA with previously published data from the mirabegron treatment group (15) as described in Methods (***P < 0.001; ****P < 0.0001). (**E** and **F**) The change (post-pre) caused by each treatment was calculated. Data indicate the mean ± SEM and were analyzed by 1-way ANOVA with previously published data from the mirabegron treatment group (15) as described in Methods (interaction *P* < 0.0001); ***P < 0.001; ****P < 0.0001.

We evaluated whether pioglitazone or combination therapy affected different factors that are proposed to modulate insulin sensitivity, including plasma levels of adiponectin, high molecular weight (HMW) adiponectin, TNF-α, and monocyte chemoattractant protein–1 (MCP-1). As expected, pioglitazone treatment significantly increased both total and HMW adiponectin, and combination treatment had a similar effect (Figure 9, A–D). This induction of total and HMW adiponectin by pioglitazone or combination treatments was significantly different from the response to mirabegron treatment, which did not increase either of these (Figure 9, E and F; *P* < 0.0001). Neither TNF-α nor MCP-1 plasma levels were changed by either pioglitazone alone or combination treatment (Table 2).

### Adipose tissue remodeling.

To examine the effect of the drug treatments on SC WAT gene expression, we measured mRNA expression using the NanoString nCounter system with a custom code set of 163 genes, as described previously (15). Pioglitazone treatment caused numerous changes in adipose gene expression (24 genes significantly changed), most of which were expected (Table 3), including adipokines and previously characterized genes involved in lipid metabolism and inflammation. Combination treatment also caused significant changes in the expression of 14 genes (Table 4), and 8 of these genes were also changed by pioglitazone treatment. We evaluated genes known to control beiging to understand the suppression of mirabegron-induced beiging by pioglitazone. TZDs have been previously shown to induce the expression of “pocket proteins,” including *P53* and *RBL1* (p107), which inhibit beiging (57–59). Our analysis indicated that *RBL1* was significantly increased by pioglitazone treatment and combination treatment (Tables 3 and 4). Induction of *RBL1* is thus a possible mechanism explaining why pioglitazone inhibited mirabegron-induced beiging when the drugs were used in combination. Although mirabegron increased adipose UCP1 levels, we previously found that mirabegron did not induce *PPARGC1A* (PGC1α) gene expression (15, 16). If pioglitazone increased *PPARGC1A* expression, this could potentially result in a synergistic effect with mirabegron. Indeed, *PPARGC1A* was significantly induced by both pioglitazone and combination treatments (Tables 3 and 4), but this did not result in additional beiging by combination treatment. We evaluated the bioenergetics profile of mitochondria purified from SC WAT as previously described (16) but did not find changes in free fatty acid–induced uncoupled respiration caused by pioglitazone or combination treatment (data not shown). However, these experiments were not performed in a way that would have revealed UCP1-dependent respiration.

### Summary and conclusions.

We hypothesized that the combination of pioglitazone and mirabegron would induce more beiging of SC WAT and/or BAT than either drug alone. Despite the fact that both drugs, when used alone, stimulated beiging, the results of this study and our previously published observations indicate that the addition of pioglitazone to mirabegron treatment resulted in less beiging of adipose than by mirabegron treatment alone. Furthermore, none of the 3 treatments induced BAT in obese research participants. This study also indicates clear differences in the mechanisms by which mirabegron and pioglitazone improve glucose homeostasis. Mirabegron treatment had unique effects on muscle, enhancing capillary density, and as previously reported inducing a switch to type I fibers and increasing plasma NEFA (15). Pioglitazone negated many of these effects on muscle and plasma NEFA yet more potently induced insulin sensitivity and increased both total and HMW adiponectin, which is an insulin sensitizer.

## Discussion

A number of clinical studies have been performed with the goal of increasing BAT or beige adipose tissue, and these studies support the concept that increasing brown and/or beige adipose tissue improves glucose and lipid homeostasis (6, 15, 17–23, 25, 34), suggesting that induction of brown and/or beige fat in humans is a strategy to combat metabolic disease. In recent studies, treatment with the β_3_AR agonist mirabegron improved glucose homeostasis in both lean and obese subjects (15, 34), and obese subjects demonstrated an increase in beige fat but no increase in BAT (15). We hypothesized that the addition of pioglitazone to mirabegron treatment would further increase beiging and/or BAT. The results of this study disprove the main hypothesis, demonstrating that pioglitazone not only inhibits mirabegron treatment–induced beiging, but other effects as well, such as increases in muscle capillary density and type I fibers and increases in plasma NEFA. Overall, this study highlights important mechanistic differences between mirabegron and pioglitazone treatment.

This study was designed to determine whether pioglitazone, a PPARγ activator, would work in an additive or synergistic manner with a β_3_AR agonist to stimulate BAT or beige fat and therefore further improve glucose and lipid metabolism. Pioglitazone induced the protein expression of the beige adipocyte markers UCP1 and TMEM26 in SC WAT, consistent with the findings from other clinical studies (46, 47) and many other studies involving rodents (40, 41) or in vitro cell culture (42–44). Despite this finding, the addition of pioglitazone to mirabegron treatment resulted in lower expression of beige adipose markers than mirabegron treatment alone. One study in vitro examining the combination of a TZD and a β_3_AR agonist indicated that maximal UCP1 expression is obtained when the TZD is used acutely and that chronic addition of the TZD inhibited the effects of the β_3_AR agonist (43). It is thus possible that there is an optimal time frame for TZD administration in combination with a β_3_AR agonist to induce beiging in vivo in humans. This study only examined abdominal SC adipose tissue, although a previous study examined the beiging response to cold and found similar changes in both femoral and abdominal SC fat (16).

Although mirabegron treatment increased SC WAT beiging, it did not increase PGC1α expression, which promotes beiging and mitochondrial biogenesis in adipose tissue (16). The ability of pioglitazone to increase *PPARGC1A* was one mechanism by which we hypothesized that combination treatment would further increase beiging. Although pioglitazone treatment, alone or in combination with mirabegron, increased *PPARGC1A* expression, it also increased *RBL1* (p107) expression, which promotes white adipose gene expression and inhibits browning (59). The induction of *RBL1* (p107) and other genes by pioglitazone is likely part of a complicated mechanism for inhibition of mirabegron-induced beiging, which could include effects of pioglitazone on mitochondria (60) or on transcription of genes not measured in our multiplex assay. Finally, we observed that addition of mirabegron to pioglitazone changed the effect of pioglitazone on gene transcription in SC WAT. This may be explained by opposing effects of the drugs on lipolysis and NEFA levels. We did not observe higher fold induction of genes significantly regulated by both treatments (e.g., *FABP4*) in the combination treatment group, even though one might expect mirabegron to cause further transactivation of PPARγ by activating p38 (61). The reasons for this observation are unclear.

Studies in rodents indicate that PPARγ is necessary for the genesis of BAT, and TZDs can increase BAT mass in rodents (39, 48, 51); however, it should be noted that those studies used considerably higher doses of TZDs than this study. Furthermore, other studies indicated synergism between PPARγ and βAR agonism with respect to thermogenesis (39, 48, 49). The induction of BAT activity in humans is more difficult than in rodents. In lean subjects, BAT can be induced by mirabegron treatment, but this is dependent on the dose of mirabegron used. Although there was some induction of BAT with a 50 mg dose in young men (35), a more robust BAT induction required higher doses, which could be due to activation of β_1_ARs (62) or β_2_ARs (63). A daily mirabegron dose of 100 mg resulted in increases in heart rate and blood pressure in lean women (34), although earlier studies of mirabegron use for overactive bladder found only minimal cardiovascular side effects at doses of 100 and 200 mg/d (64, 65). Obese subjects have less BAT than lean subjects, and neither mirabegron at 50 mg/d (15), nor pioglitazone, alone or in combination with mirabegron, successfully induced BAT in this cohort of persons with obesity. These results suggest that pharmacological induction of BAT in obese humans will be challenging. Notably, in the 3 subjects who demonstrated BAT at baseline, pioglitazone treatment decreased BAT volume. Although more studies of subjects with BAT would be needed to draw a definitive conclusion, this result is consistent with a recent study demonstrating that pioglitazone treatment decreased BAT induction by cold in lean humans (66). Some studies have successfully induced BAT in obese subjects and reported improvement in metabolic homeostasis using cold as the stimulus, suggesting that βAR agonism may be utilized to induce BAT (20, 22). More specific β_3_AR agonists could be tested in the future to avoid cardiovascular side effects. Alternatively, capsinoids, which are cold mimetics that are effective in lean subjects (19), could be evaluated in future studies.

We found several important differences between mirabegron and pioglitazone treatment in skeletal muscle that suggest a potential mechanism for the differences in insulin sensitivity response between the treatments. Mirabegron increased type I fibers in vastus lateralis biopsies (15) and increased muscle capillary density (Figure 7). Since type I fibers have more capillaries (55, 56), these changes in muscle fiber type and capillarization may be related and part of the mechanism by which mirabegron increased insulin sensitivity. However, pioglitazone decreased both type I fibers and capillary density. Fiber type switching has not been extensively studied in humans in response to TZD treatment. One paper found no change in fiber type in subjects with diabetes after troglitazone treatment (67). Similarly, muscle capillary density has not been studied in humans in response to pioglitazone. Notably, the effects of mirabegron on muscle fiber type and capillary density were inhibited by pioglitazone when it was added to mirabegron treatment. Interestingly, the effects of the drug treatments on muscle followed the same pattern as the effects of the drug treatments on plasma NEFA levels. This raises the possibility the plasma NEFA levels contribute to the regulation of muscle fiber type by mirabegron treatment in obese humans by stimulating the switch to more oxidative and insulin-sensitive type I fibers. Finally, pioglitazone potently stimulated the insulin-sensitizing adipokine adiponectin (total and HMW) whereas mirabegron did not. Overall, these results indicate different mechanisms for increasing insulin sensitivity by mirabegron and pioglitazone. Mirabegron treatment stimulated WAT beiging, lipolysis, and plasma NEFA (15). Whereas elevated NEFA is often associated with insulin resistance, mirabegron treatment increased muscle type I fibers and capillary density, resulting in improved insulin sensitivity. These effects may be due to NEFA stimulation of PPAR transcription factors in muscle to increase *PPARGC1A* (PGC1α), which we previously observed (15). Another possibility is that mirabegron causes β_3_AR-expressing cell types to secrete a factor(s) that acts on muscle. Pioglitazone, on the other hand, decreased NEFA and stimulated insulin sensitivity without changes in muscle fiber type, which may be explained by the increase in the antiinflammatory and insulin-sensitizing adipokine adiponectin.

A potential limitation of this study was the quantification of adipose beiging using immunohistochemistry. Although we always performed staining of the pre and post treatment biopsies at the same time, the immunohistochemistry was not all performed together. Thus, baseline expression levels may have been influenced by experimental variables, such as differences in lot number for the antibodies or other reagents used to quantify beige marker protein expression. Furthermore, we have detected UCP1 expression in cells other than beige adipocytes, such as vascular cells (16) and macrophages (15), which may have a small effect on the quantification of beiging. Despite these limitations, we have observed consistent induction of the 3 beige markers in SC WAT in response to cold and β_3_AR stimulation with mirabegron (15, 16).

In summary, contrary to our original hypothesis, pioglitazone cannot be added to mirabegron treatment to increase either beiging or BAT volume. This study revealed several important differences between pioglitazone and mirabegron treatment in their effects on muscle fiber type and vascularity, suggesting that plasma NEFA levels are involved.

## Methods

### Study design and human subjects.

This study was designed to evaluate the effect of combining pioglitazone and mirabegron on SC WAT beiging, BAT volume and activity, glucose homeostasis, and underlying mechanisms. Combination treatment was thus compared with the effect of each drug alone by analyzing the change (post-pre) caused by each of the 3 treatments. Data from the mirabegron arm of the trial was calculated using recently published data (15). The research participants in the pioglitazone and combination treatment arms of the trial were recruited and analyzed during the same time frame as those in the mirabegron arm of the trial using the same criteria (15). Briefly, subjects were recruited who were sedentary, were 35 to 65 years old, had a BMI more than 27, and either had prediabetes or were normal glucose tolerant with 3 features of metabolic syndrome based on fasting labs and a standard 75 g OGTT. The research participants self-identified their race, sex, and other demographic information, and data are provided for the entire cohort. Procedures performed at baseline included a DEXA for body composition, resting metabolic rate using a metabolic cart, PET CT scan for BAT assessment using the protocol described below, a euglycemic clamp for measurement of peripheral insulin sensitivity, and SC WAT and vastus lateralis biopsies, all of which were described previously (15). Subjects were randomized to receive mirabegron (50 mg), pioglitazone (30 mg), or both drugs daily for 12 weeks. The drugs were purchased from the hospital pharmacy, and this was an open-label study.

### BAT assessment.

We assessed BAT activity using methods described previously (68). After an overnight fast, subjects were brought to the PET CT suite and outfitted with a cooling vest that circulated water at 14°C. Subjects were asked to report shivering, but this occurred only rarely. If shivering was reported, the water temperature was increased by 1°C and shivering stopped. The cooling occurred for 60 minutes prior to i.v. injection of 10–20 mCi (370–740 megabecquerel) fluorodeoxyglucose (^18^F-FDG) and then for another 60 minutes after tracer injection, and prior to scanning. PET CT images were then acquired as outlined in Supplemental Methods. In brief, subjects’ weights and heights were measured, and activity of the syringe pre- and postinjection was measured to calculate net injected dose. Sixty minutes after ^18^F-FDG injection, CT and PET images of the torso from the eyes to the thighs were acquired and reconstructed as axial sections and reformatted by the viewer software. Regions of ^18^F-FDG uptake on PET that colocalized with areas of fat based on Hounsfield units identified by CT were quantitated by calculating the standardized uptake value average, and BAT volume was calculated as the sum of areas meeting these characteristics.

### Immunohistochemistry.

Immunohistochemistry on UCP1, TMEM26, and CIDEA in SC WAT was performed using the following primary antibodies: custom UCP1 antibody (ECM Biosciences catalog J2648), TMEM26 (NBP2-27334, Novus Biologicals), and CIDEA (H00001149-M01, Novus Biologicals) as described previously (15). We did not quantify staining in fibrotic areas. Sections from the same subject (pre and post drug treatment) were always stained at the same time, and images were obtained using similar exposure times for all sections. The area stained was assessed using a thresholding feature of Zeiss software (Zen) and was normalized to adipocyte number (unilocular adipocytes) since human subjects demonstrate a range of adipocyte sizes. Briefly, analysis of sections taken from the fat biopsies immunoreacted with UCP1, TMEM26, or CIDEA was performed as follows. Images were taken using a fluorescence camera to capture Texas red fluorescence. Zen Pro2 software (Zeiss) was used for image acquisition, and images were then subjected to the thresholding feature in the Zen Imaging Wizard using set parameters for the specific intensity of the Texas red fluorescence. All areas reaching the threshold were outlined and sum of all areas is reported as square micrometers. For each image, the number of cells was counted and the total thresholded area was normalized to cell number. Zen Blue image analysis software (Zeiss) was used to automatically measure the size of adipocytes using autofluorescence to outline adipocytes in images taken at 10× magnification. Muscle fiber typing was done as described (15). Briefly, frozen muscle sections were stained with isotype-specific antibodies (Developmental Studies Hybridoma Bank) against MyHC I IgG2B (BA.D5), MyHC IIa IgG1 (SC.71), and MyHC IIx (6H1) followed by isotype-specific secondary antibodies: goat anti-mouse IgG2b Alexa Fluor 647 (A21242, Invitrogen, Thermo Fisher Scientific), goat anti-mouse IgG1 Alexa Fluor 488 (A21121, Invitrogen, Thermo Fisher Scientific), and goat anti-mouse IgM Alexa Fluor 555 (A21426, Invitrogen, Thermo Fisher Scientific). Muscle capillary density was determined by staining muscle sections with TRITC-conjugated Lectin from Ulex europaeus UEA-1 (MilliporeSigma, L4889) at a dilution of 1:50. Sections were analyzed by blinded assessors.

### Plasma NEFA, glycerol, adiponectin, and cytokine measurements.

Plasma NEFA levels were determined with HR Series NEFA-HR(2) Color Reagents (999-34691, 995-34791, 991-34891, 993-35191, FUJIFILM Wako Diagnostics). Plasma glycerol was measured with Free Glycerol Reagent (F6428, MilliporeSigma). Plasma total and HMW adiponectin were measured by an ELISA kit (ALPCO 80-ADPHU-E01). Plasma TNF-α and MCP-1 were measured using custom multiplex cytokine assays (Meso Scale; C4049/CP000079).

### Muscle lipids.

Diacylglycerides (DAGs), ceramides, and TGs were quantified in the muscle biopsies as described (15). Briefly, approximately 10 mg of the vastus lateralis biopsy was weighed and then extracted with acidified organic solvents. DAGs and ceramides were quantified in the extracts as described previously (69). TGs were quantified in the extract using colorimetric TG assays (T7532, Point Scientific) as described (15).

### mRNA quantification.

Multiplex analysis of SC WAT gene expression was performed as described previously (15). mRNA from SC WAT was isolated using TRIzol extraction and purification with RNeasy Lipid Tissue Mini Kits (QIAGEN). Gene expression was then quantified using the NanoString nCounter system and a custom code set. The custom code set and normalization were performed as described (15).

### Statistics.

The study was initially powered to include 20 subjects in each trial arm to detect as little as a 25% increase in beiging with 80% power. We ended the trial with 12–13 subjects in each trial arm since we were able to detect significant changes between treatment groups in the primary and several secondary endpoints. In order to determine whether the responses to each of the 3 drug treatments were different, we performed 1-way ANOVAs on change scores using SAS version 9.4. In some instances, the results of each individual drug treatment were analyzed by paired, 2-tailed Student’s *t* tests using GraphPad Prism version 8.0. Oral glucose tolerance was assessed by repeated measures 2-way ANOVA with a Sidak’s multiple-comparison test using GraphPad Prism. The NanoString assay is a highly reproducible assay that was highly targeted to genes important for adipose tissue function, and we did not correct for multiple comparisons. The method of analysis for each experiment is indicated in the figure legend. All tests were 2 sided, statistical significance was set at *P* ≤ 0.05, and statistical trend was set at *P* ≤ 0.10.

### Study approval.

All subjects gave written informed consent, and the protocols were approved by the Institutional Review Board at the University of Kentucky.

## Author contributions

PAK, EEDV, PGS, AJM, and BSF designed the experiments, analyzed data, and wrote the manuscript. BZ, HM, ALC, RHEK, JC, ZRJ, and HJV performed the experiments. PMW analyzed data. The order of first authors was determined alphabetically.

## Supplementary Material

Supplemental data

## Figures and Tables

**Table 4:**
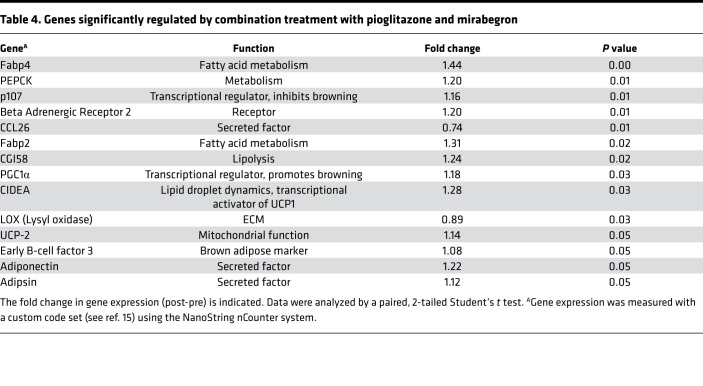
Genes significantly regulated by combination treatment with pioglitazone and mirabegron

**Table 3:**
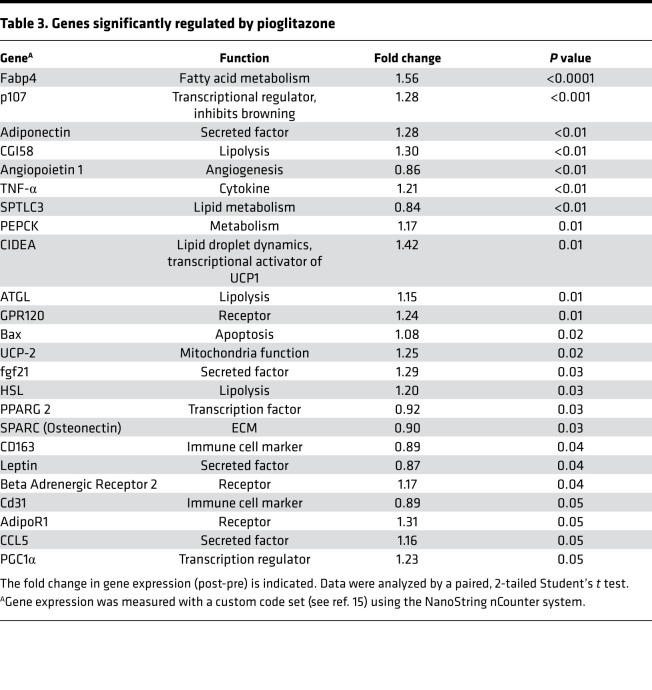
Genes significantly regulated by pioglitazone

**Table 1:**
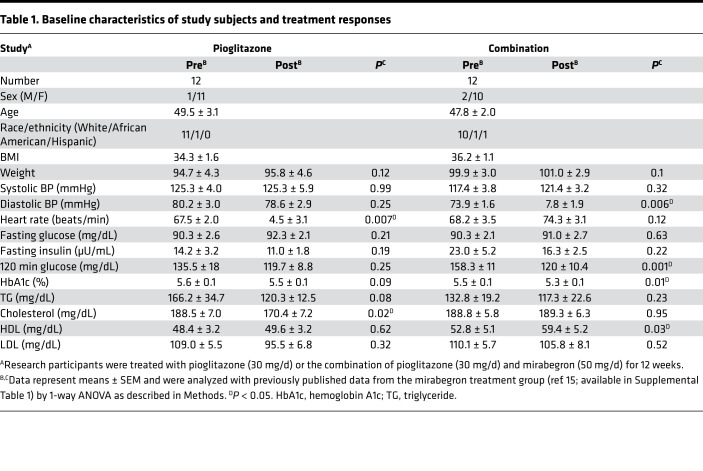
Baseline characteristics of study subjects and treatment responses

**Table 2:**
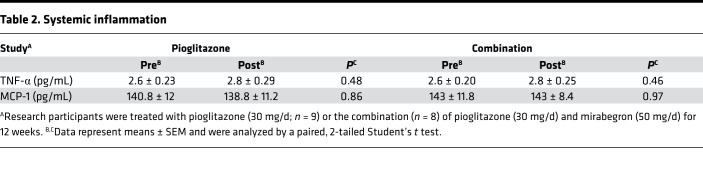
Systemic inflammation

## Version 1 02/11/2021

In-Press Preview

## Version 2 03/22/2021

Electronic publication
