# Supplementary material for: Pioglitazone does not synergize with mirabegron to increase beige fat or further improve glucose metabolism
Source: JCI Insight. 2021 Mar 22;6(6):e143650. doi: 10.1172/jci.insight.143650 (PMC8026187; doi:10.1172/jci.insight.143650)
Supplement: Supplemental data [file jciinsight-6-143650-s031.pdf]

Supplemental Methods

Detailed description of PET-CT methods. All research participants included in the study had scans performed on a Siemens Biograph TruePoint 6-slice PET/CT (Siemens Healthcare, Erlangen, Germany). Subjects' weights and heights were measured and recorded in our PET/CT center prior to radiopharmaceutical injection. The scan protocol was developed according to the Society of Nuclear Medicine and Molecular Imaging guidelines. Activity of the syringe pre- and post-injection were measured to calculate the net injected dosage for each research participant. Sixty minutes after intravenous administration of 10-20 mCi (370-740 MBq) ^{18}F-FDG activity, CT and PET images of the torso from skull-base to mid-thigh were acquired with the subject in the supine position and arms placed above the head. Subjects were asked to void prior to scan acquisition. CT images were acquired without intravenous or oral iodinated contrast administration with the following parameters: reference tube voltage of 130 kVp, reference tube current 88 mAs and online dose modulation (CARE Dose 4D, Siemens), spiral pitch factor 1.5, field of view 50 x 50 cm, and matrix 512 x 512; 5 mm slices were reconstructed using a standard soft tissue reconstruction kernel (standard filtered back projection B30s). Following CT acquisition 3D PET images were acquired with the following parameters: acquisition time of 4 minutes per table position, 168 x168 matrix, and 50 x 50 cm field of view. After decay and scatter correction, PET data were reconstructed using ordered-subsets expectation maximization with 2 iterations and 8 subsets using the algorithm implemented by the manufacturer (esoft, Siemens, Erlangen, Germany). All images were corrected for attenuation using the co-registered CT scan.

PET/CT Image Analysis was performed as follows. Multi-planar reconstruction of PET and CT datasets resulted in fused PET/CT images in axial, sagittal and coronal planes. PET/CT scans were reviewed and analyzed by a single reader using dedicated PET/CT image analysis software (Mirada version XD_3, Mirada Medical Ltd, New Road, Oxford, UK).

Standardized Uptake Values were determined as follows. Multiple 3D volume of interest (VOI) were placed within activated brown fat regions including but not limited to in the cervical, supraclavicular, and anterior thoracic regions. The SUV calculation is based on the standard equation(68):

$$\frac{\boldsymbol{Activity\ concentration\ in\ tissue}}{\boldsymbol{Administered\ activity/body\ size}}$$

Administered activity is the result of (Pre-injection syringe activity – post-injection residual activity), and multiple measure can be used for body size normalization such as body weight, lean body mass, body surface area, among others. In this study we focused on body weight and lean body mass normalized SUVs only. From each VOI, the mean and maximum values of SUV_{bw} and SUL were extracted. Hounsfield Unit measurements were performed using the unenhanced CT part of the PET/CT acquisition. Regions of interest were placed within the activated brown fat regions and compared to the right lobe of the liver.

Table S1. Baseline characteristics of mirabegron treatment study subjects and treatment responses.

	Mirabegron[a] Pre	Post	P-value[b]
**Number (Gender M/F)**	13 (2/11)		
**Age (years)**	54.8 ± 2.2		
**BMI (kg/m^2)**	32.4 ± 1.1		
**Weight (kg)**	88.0 ± 2.6	87.5 ± 2.6	0.34
**Systolic BP (mmHg)**	127.8 ± 4.8	125.5 ± 3.5	0.42
**Diastolic BP (mmHg)**	79.8 ± 2.3	81.4 ± 2.6	0.61
**Heart Rate (beats / min)**	68.7 ± 1.6	70.7 ± 1.8	0.32
**Fasting Glucose (mg/dl)**	97.0 ± 2.8	95.8 ± 3.1	0.49
**Fasting Insulin (μU/mL)**	13.2 ± 2.7	14.4 ± 2.3	0.4
**120 min Glucose (mg/dl)**	135.3 ± 7.5	109.8 ± 6.4	<0.01
**HbA1c (%)**	5.6 ± 0.1	5.4 ± 0.1	0.01
**TG (mg/dl)**	144.0 ± 22.9	130.1 ± 18.0	0.35
**Cholesterol (mg/dl)**	215.7 ± 11.2	205.7 ± 11.6	0.09
**HDL (mg/dl)**	56.6 ± 5.1	55.9 ± 4.7	0.71
**LDL (mg/dl)**	132.9 ± 7.8	125.4 ± 8.6	0.15

[a]Research participants were treated with mirabegron (50 mg/day) for 12 weeks. Data represent mean ± SEM (n=13). [b]Data were analyzed by paired, two-tailed Student's t-test. This table was previously published (15).

Table S2. Results from DEXA.

Study[a]	Pioglitazone			Combination		
	Pre[b]	Post[b]	P[c]	Pre[b]	Post[b]	P[c]
Total Fat (kg)	42.4 ± 3.2	43.6 ± 3.4	0.03*	47.0 ± 2.4	47.7 ± 2.3	0.84
Total Lean (kg)	49.1 ± 2.0	49.3 ± 2.1	0.73	49.4 ± 2.1	49.8 ± 2.1	0.76

[a]Research participants were treated with pioglitazone (30 mg/day) or the combination of pioglitazone (30 mg/day) and mirabegron (50 mg/day) for 12 weeks. [b,c]Data represent means ± SEM (n=12) and were analyzed by a paired, two-tailed Student's t-test.

Table S3. Results from Indirect Calorimetry.

Study[a]	Pioglitazone Pre[b]	Pioglitazone Post[b]	P[c]	Combination Pre[b]	Combination Post[b]	P[c]
VO_2	0.236 ± 0.010	0.230 ± 0.010	0.14	0.255 ± 0.014	0.246 ± 0.010	0.39
VCO_2	0.199 ± 0.010	0.194 ± 0.010	0.35	0.202 ± 0.008	0.195 ± 0.010	0.36
RQ	0.841 ± 0.013	0.843 ± 0.019	0.91	0.803 ± 0.025	0.795 ± 0.023	0.70
REE	1654 ± 75	1611 ± 70	0.2	1765 ± 84	1701 ± 69	0.30

[a]Research participants were treated with pioglitazone (30 mg/day) or the combination of pioglitazone (30 mg/day) and mirabegron (50 mg/day) for 12 weeks. [b,c]Data represent means ± SEM (n=12) and were analyzed by a paired, two-tailed Student’s t-test.

Table S4. Analysis of the change in treatment responses.

	Treatment			
**Change in**	Mirabegron	Pioglitazone	Combination	P[a]
**Weight (kg)**	-0.54 ± 0.53	1.15 ± 0.68	0.91 ± 0.51	0.10
**Fasting glucose (mg/dl)**	-1.23 ± 1.72	2.0 ± 1.53	0.75 ± 1.50	0.39
**Fasting insulin (μU/ml)**	1.04 ± 1.19	-3.18 ± 2.22	-6.70 ± 4.74	0.12
**HbA1c (%)**	-0.17 ± 0.06	-0.1 ± 0.05	-0.22 ± 0.07	0.41
**TG (mg/dl)**	-13.9 ± 14.4	-45.9 ± 23.1	-15.4 ± 12.2	0.47
**Cholesterol (mg/dl)**	-10.0 ± 5.4	-18.2 ± 6.3	0.41 ± 5.9	0.12
**HDL (mg/dl)**	-0.7 ± 1.8	1.3 ± 2.5	6.6 ± 2.6	0.10
**LDL (mg/dl)**	-7.5 ± 5.05	-13.5 ± 5.3	-4.3 ± 6.5	0.53
**Disposition index (insulinogenic * GIR high glucose)**	3.02 ± 0.99	2.27 ± 1.54	0.51 ± 0.67	0.13

[a]The change (post-pre) was calculated for each treatment. Data used to calculate the change caused by mirabegron treatment were previously published (15). Data were analyzed by ANOVA as described in Methods. The interaction P value is indicated.

Table S5. Results from PET-CT scans

Study[a]	Pioglitazone			Combination		
	Pre[b]	Post[b]	P[c]	Pre[b]	Post[b]	P[c]
SUV_{mean}	0.78 ± 0.22	0.74 ± 0.20	0.18	0.72 ± 0.24	0.94 ± 0.3	0.48
SUV_{max}	1.33 ± 0.41	1.15 ± 0.35	0.54	1.41 ± 0.58	1.28 ± 0.44	0.83
SUV_{peak}	1.11 ± 0.34	0.92 ± 0.28	0.39	1.09 ± 0.44	1.02 ± 0.35	0.88
Glycolysis	67.0 ± 44.5	14.3 ± 7.1	0.21	80.0 ± 51.0	107.9 ± 67.4	0.71

[a]Research participants were treated with pioglitazone (30 mg/day) or the combination of pioglitazone (30 mg/day) and mirabegron (50 mg/day) for 12 weeks. [b,c]Data represent means ± SEM (n=10) and were analyzed by a paired, two-tailed Student's t-test.

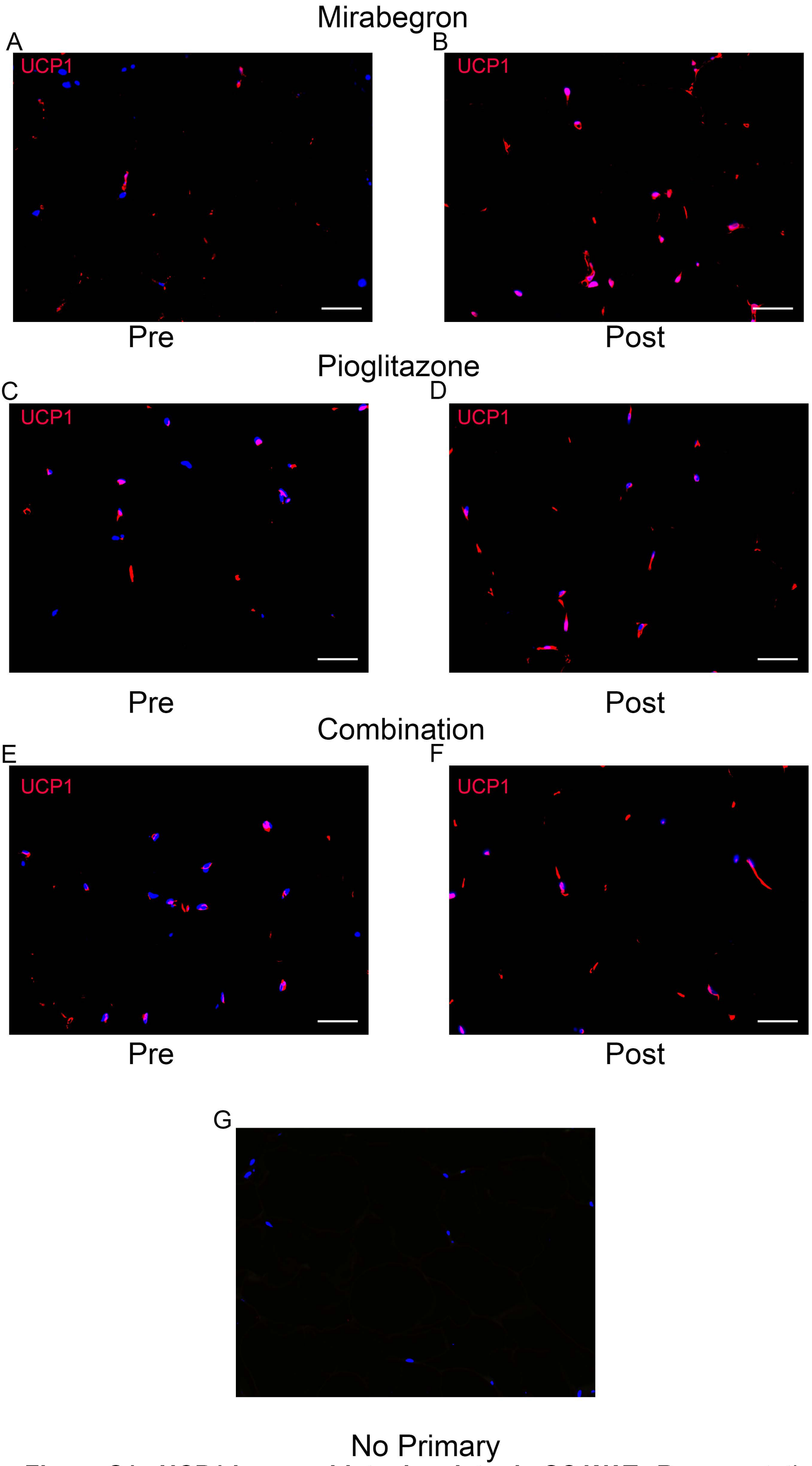

Figure S1. UCP1 immunohistochemistry in SC WAT. Representative images of UCP1 staining in SC WAT are shown; a subject with a change in UCP1 close to the average was selected (n=12 to 13; see Figure 3). A and B) images are before and after mirabegron treatment as indicated. C and D) images are before and after pioglitazone treatment as indicated. E and F) images are before and after combination treatment as indicated. G) No primary antibody control. UCP1 staining is indicated in red; nuclei staining with DAPI is indicated in blue; scale bars: 50 μm.

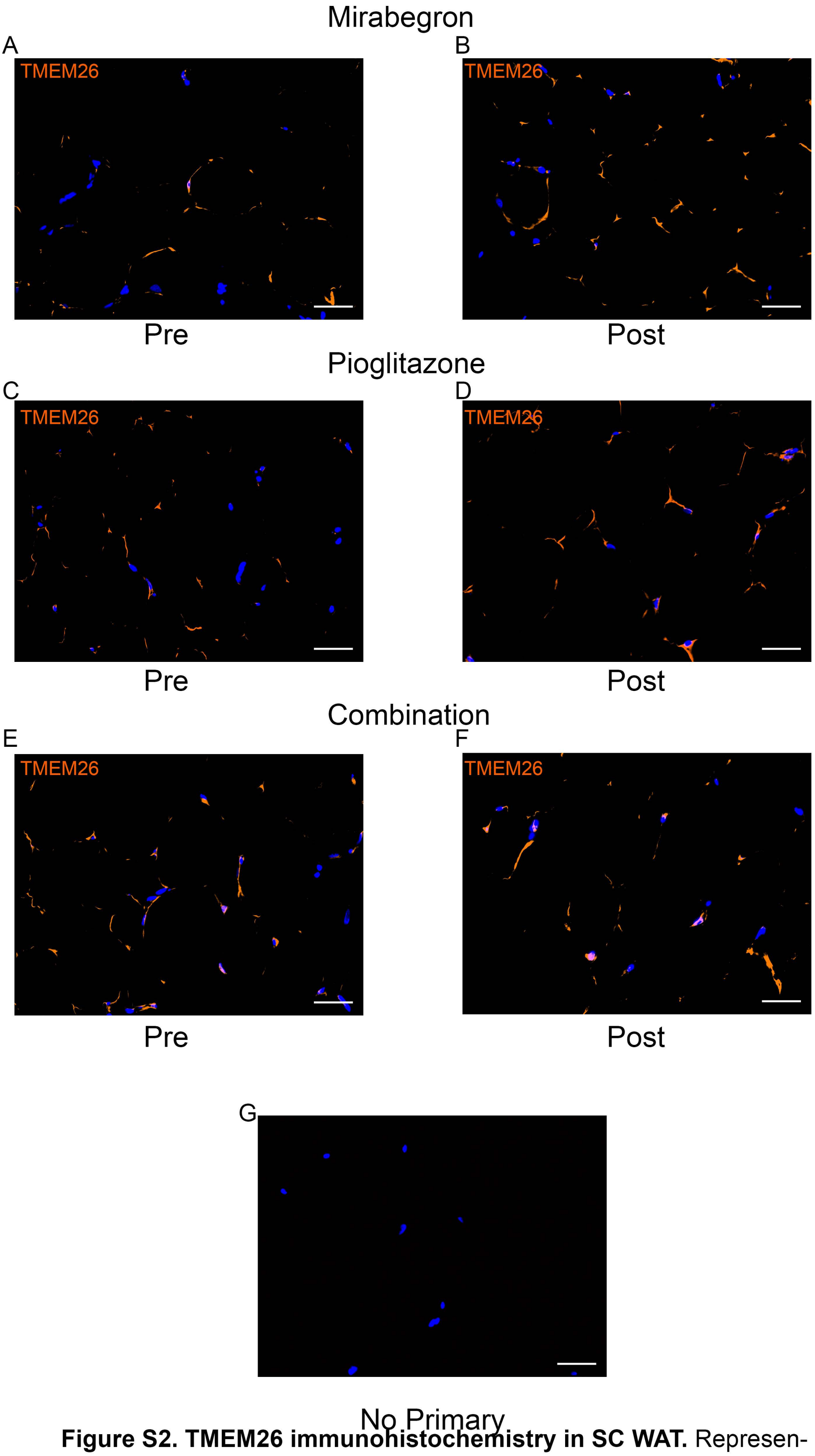

Figure S2. TMEM26 immunohistochemistry in SC WAT. Representative images of TMEM26 staining in SC WAT are shown; a subject with a change in TMEM26 close to the average was selected (n=12 to 13; see Figure 3). A and B) images are before and after mirabegron treatment as indicated. C and D) images are before and after pioglitazone treatment as indicated. E and F) images are before and after combination treatment as indicated. G) No primary antibody control. TMEM26 staining is indicated in orange; nuclei staining with DAPI is indicated in blue; scale bars: 50 μm.

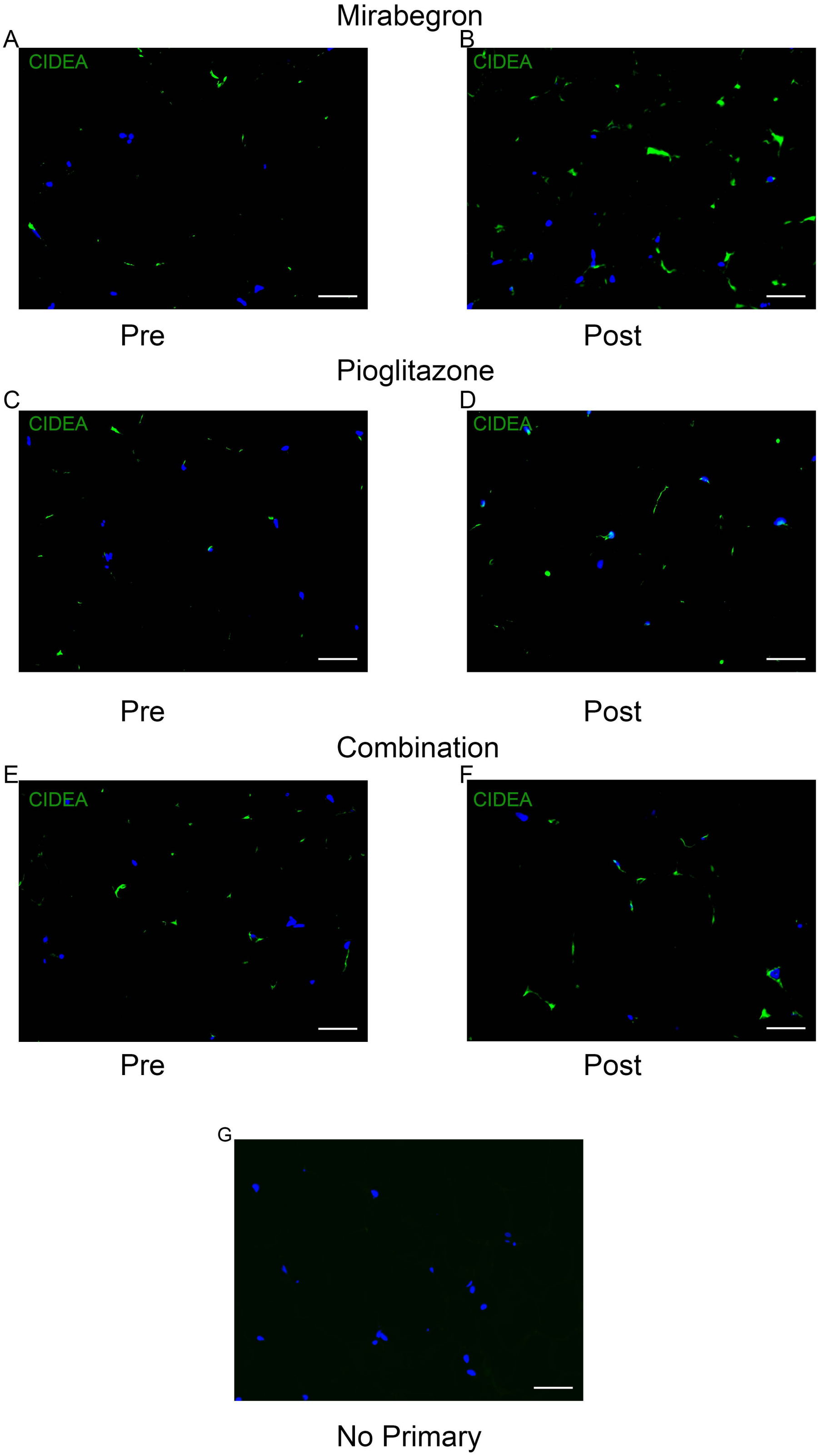

Figure S3. CIDEA immunohistochemistry IN SC WAT. Repesentative images of CIDEA staining in SC WAT are shown; a subject with a change in CIDEA close to the average was selected (n=12 to 13; see Figure 3). A and B) images are before and after mirabegron treatment as indicated. C and D) images are before and after pioglitazone treatment as indicated. E and F) images are before and after combination treatment as indicated. G) No primary antibody control. CIDEA staining is indicated in green; nuclei staining with DAPI is indicated in blue; scale bars: 50 μm.

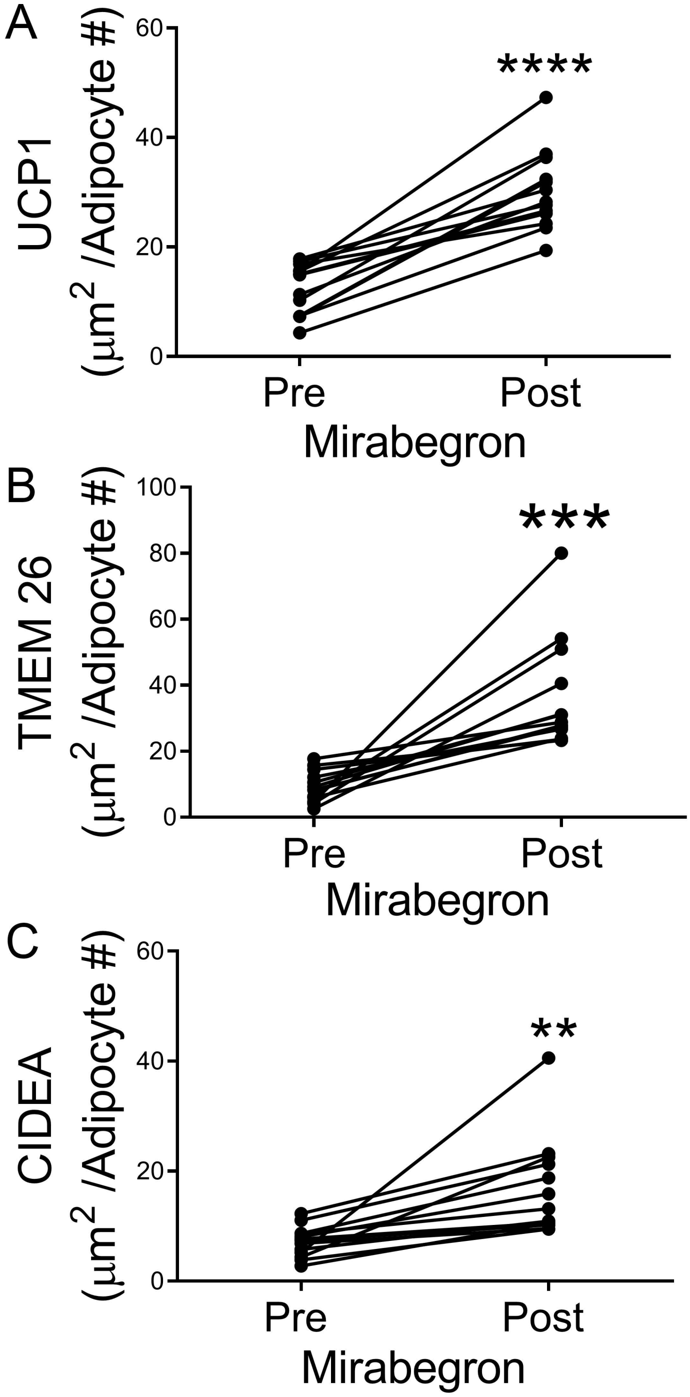

Figure S4. Quantification of the effect of mirabegron on beige marker protein expression in SC WAT. SC WAT was isolated before and after mirabegron treatment, and UCP1 (A), TMEM26 (B), and CIDEA (C) were analyzed by immunohistochemistry and quantified (n=13). Data were analyzed by a paired, two-tailed Student's t test. The data has been previously published (15, 16).

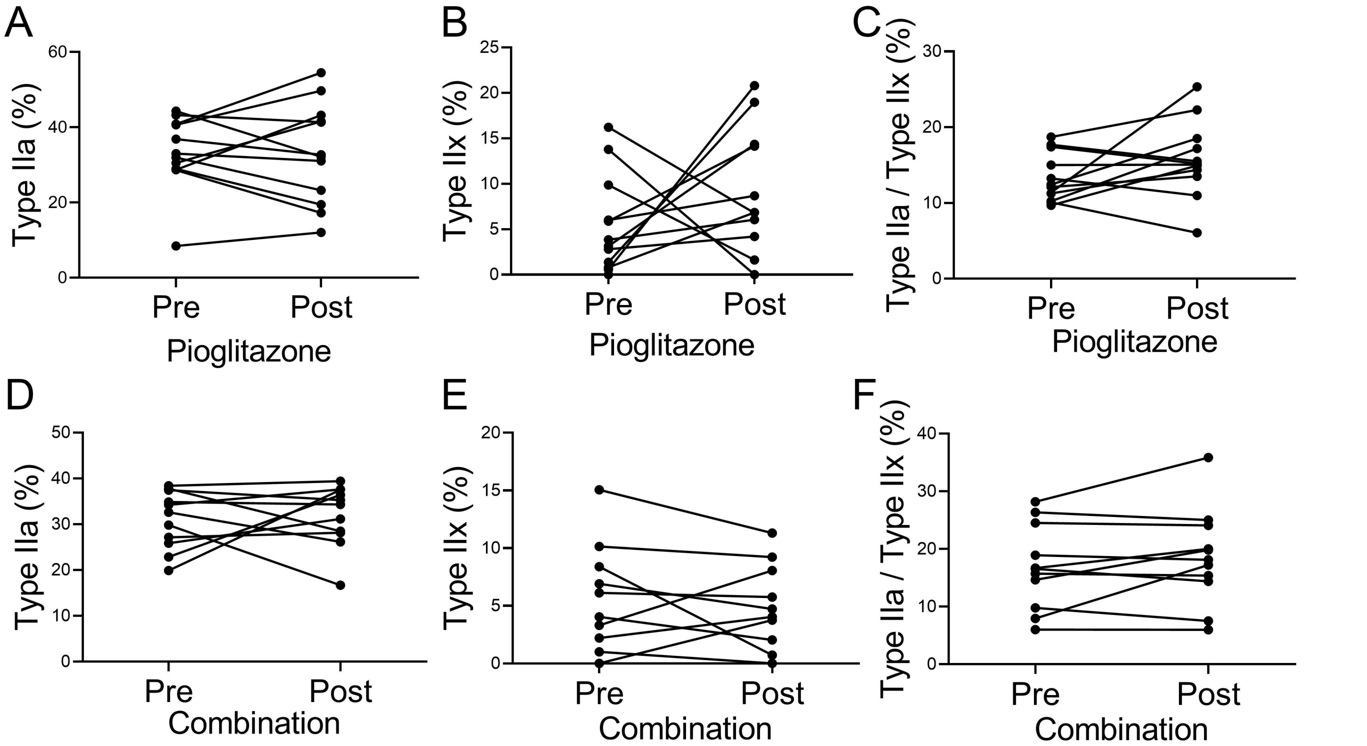

Figure S5. Muscle fiber type composition. The vastus lateralis biopsies were stained for type IIa and type IIx fibers as described in Methods. A through C) the percentage of type IIa, type IIx, and type IIa /type IIx fibers before and after pioglitazone treatment (n=12). D through F) the percentage of type IIa, type IIx, and type IIa /type IIx fibers before and after combination treatment (n=11). Data were analyzed by a paired, two-tailed Student's t test.

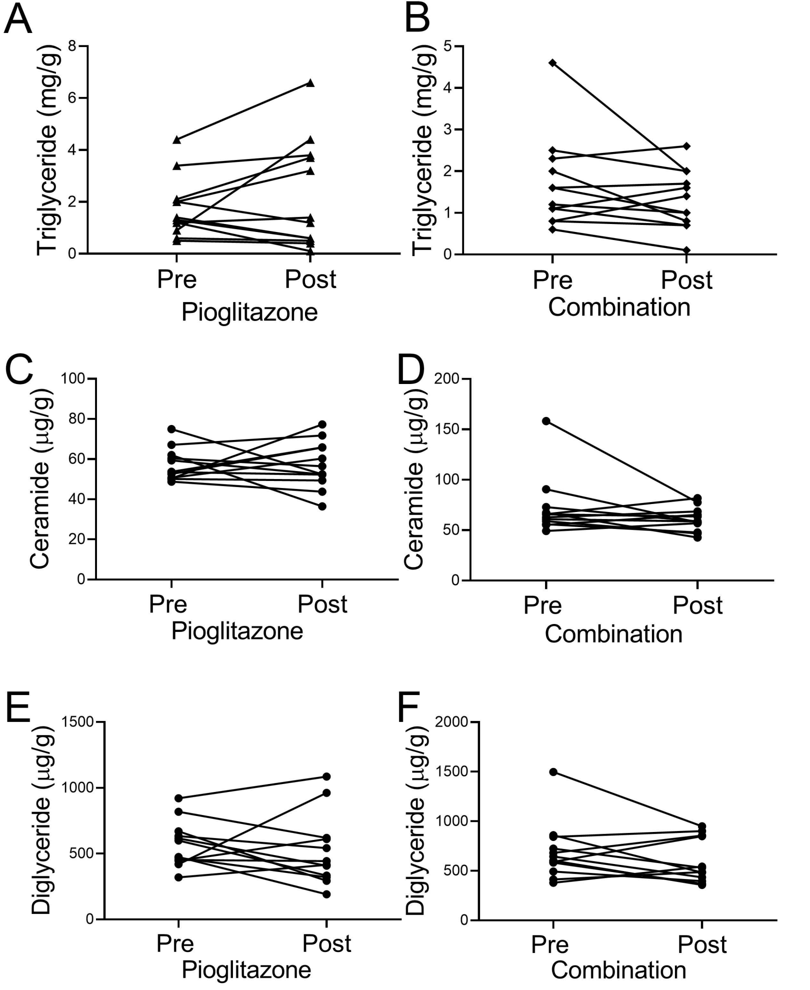

Figure S6. Muscle lipids. Lipids were extracted from the vastus lateralis biopsies and measured as described in Methods. A) and B) The levels of triglycerides before and after pioglitazone or pioglitazone and mirabegron treatment are shown. C) and D) The levels of ceramides before and after pioglitazone or pioglitazone and mirabegron treatment are shown. E) and F) The levels of diglycerides before and after pioglitazone or pioglitazone and mirabegron treatment are shown. Data were analyzed by a paired, two-tailed Student's t test (n=12).
